# Molecular Mechanism Behind the Capture of Fluorinated Gases by Metal–Organic Frameworks

**DOI:** 10.1007/s40820-024-01584-1

**Published:** 2025-01-27

**Authors:** Qian Wang, Yong Hu, Yifan Gu

**Affiliations:** 1 College of Environmental Science and Engineering, State Key Laboratory of Pollution Control and Resource Reuse, Tongji University, Siping Rd 1239, Shanghai, 200092 People’s Republic of China; 2 Department of Polymeric Materials, School of Materials Science and Engineering, Tongji University, Caoan Road 4800, Shanghai, 201804 People’s Republic of China; 3 Shanghai Institute of Pollution Control and Ecological Security, Shanghai, 200092 People’s Republic of China; 4 Key Laboratory of Cities’ Mitigation and Adaptation to Climate Change, China Meteorological Administration (CMA), Tongji University, Shanghai, 200092 People’s Republic of China

**Keywords:** Fluorinated gas, Metal–organic framework, Adsorption, Separation, Molecular interaction

## Abstract

Fluorinated gases (F-gases) play a vital role in the chemical industry and in the fields of air conditioning, refrigeration, health care, and organic synthesis. However, the direct emission of waste gases containing F-gases into the atmosphere contributes to greenhouse effects and generates toxic substances. Developing porous materials for the energy-efficient capture, separation, and recovery of F-gases is highly desired. Recently, as a highly designable porous adsorbents, metal–organic frameworks (MOFs) exhibit excellent selective sorption performance toward F-gases, especially for the recognition and separation of different F-gases with highly similar properties, showing their great potential in F-gases control and recovery. In this review, we discuss the capture and separation of F-gases and their azeotropic, near-azeotropic, and isomeric mixtures in various application scenarios by MOFs, specifically classify and analyze molecular interaction between F-gases and MOFs, and interpret the mechanisms underlying their high performance regarding both adsorption capacity and selectivity, providing a repertoire for future materials design. Challenges faced in the transformation research roadmap of MOFs adsorbent separation technologies toward F-gases are also discussed, and areas for future research endeavors are highlighted.

## Highlights

- The progress of metal–organic frameworks (MOFs) in capturing and separating F-gases is highlighted.
- The molecular mechanisms of adsorption and separation are classified and analyzed.
- Toolboxes of MOFs structural design for fluorinated gases separation are provided.

## Graphical abstract

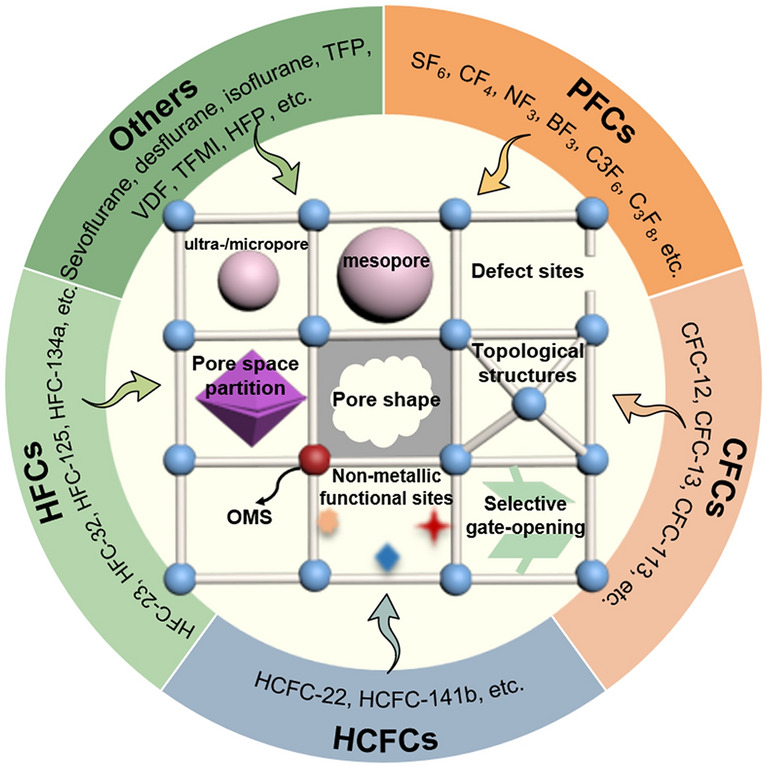

## Introduction

**Fig. 1:**
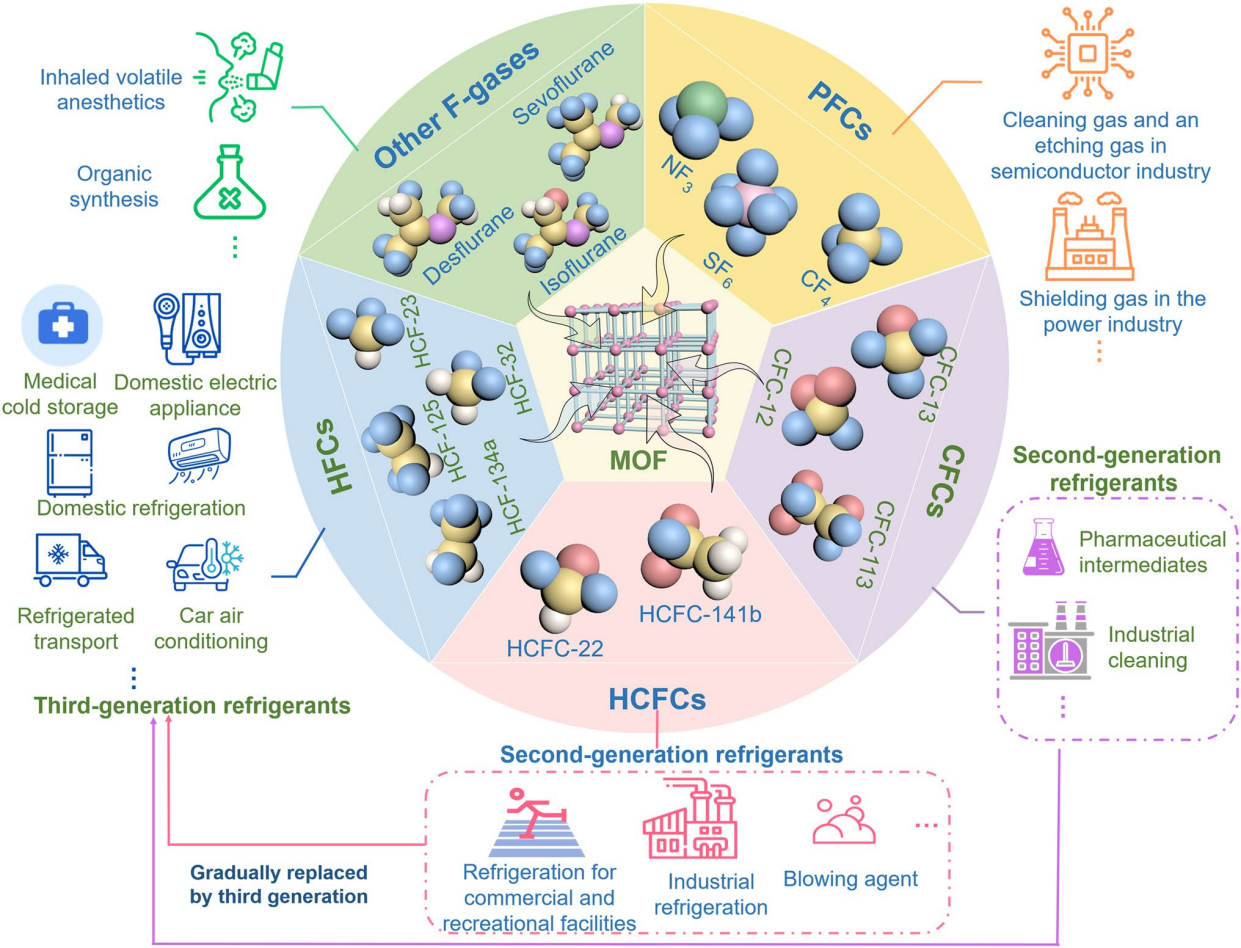
Schematic diagrams for trapping typical F-gases in a MOF platform

Fluorinated gases (F-gases), including perfluorocarbons (PFCs), hydrofluorocarbons (HFCs), chlorofluorocarbons (CFCs), hydrochlorofluorocarbons (HCFCs), inhaled volatile anesthetics (VAs), and other fluorine-containing gaseous compounds (Fig. 1), are widely used in air conditioning, refrigeration, medical, cable, semiconductor, and metal-processing industries [1–4]. The increasing use and emissions of F-gases have raised widespread environmental concerns. Most F-gases are typical greenhouse gases that exhibit much higher global warming potential (GWP) and longer atmospheric lifetime than carbon dioxide (CO_2_) and methane (CH_4_), and even lead to the depletion of the ozone layer [5–8]. In recent years, the greenhouse effect resulting from F-gases and related substances (such as carbon tetrachloride) emitted annually by various industries is equivalent to emitting 870 million tonnes of CO_2_ equivalent, which is comparable to that of over 200 coal-fired power plants [9, 10]. In addition, the emissions and hydrolysis of F-gases are often accompanied by toxic or corrosive concomitants, such as HF, CO, and NO_x_, which further adversely affect air quality and human health [11–16]. Therefore, achieving the effective capture, separation and recovery of F-gases to realize the climate goals, and the sustainable development of corresponding industries is top priority.

To date, cryogenic distillation is the main technology for separating F-gases for industrial applications [17–21]. However, some components of F-gases blends are similar in thermophysical properties, exhibiting azeotrope or near-azeotrope. For example, the boiling points of NF_3_ and CF_4_ differ by approximately 1 K (143.35 K for NF_3_ and 144.95 K for CF_4_) and their relative volatility less than 1.05 at 193.15 K. Thus, cryogenic distillation is difficult to perform [22–25]. In addition, the huge energy consumption and anticorrosion equipment design of cryogenic distillation render it an infeasible choice [17, 23]. Membrane-based separation technologies are hampered by their low intrinsic permeability to F-gases and have low separation efficiency [23, 26–30]. Moreover, they have limited applications to large-scale membrane preparation and mass membrane production [31, 32]. Physical adsorptive separation based on porous materials has high selectivity, simple operation, and easy recovery, emerging as an energy-saving and environmentally friendly option for F-gases treatment [33–39]. However, traditional porous materials, such as zeolites and carbon materials, are highly stable but have poor separation selectivity toward F-gases [40–44]. Some F-gases (such as CHClF_2_) can react chemically with zeolites, and this feature is not conducive to desorption and sorbent recycling [45–47]. Moreover, in the separation of typical F-gases/N_2_ mixed gases, negatively charged oxides on zeolite surfaces and isolated cations on the front surfaces can enhance interactions with nitrogen molecules, which decrease selectivity to F-gases [40]. Therefore, designing efficient adsorbents for F-gases separation is highly desirable.

Metal–organic frameworks (MOFs) or porous coordination polymers are crystalline porous materials with periodic network structures formed by metal ions, clusters, and organic ligands through self-assembly [48–54]. As a highly designable porous material with multiple dimensions and customizable channels, MOFs have unique features for selectively adsorbing targets and have been widely used in olefin or alkane separation [55–58]; benzene, cyclohexane, and xylene separation [59–63]; carbon capture [64–69]; and natural gas purification [70–74]. Although research on MOFs for F-gases capture and purification is in the early stage, it has fully demonstrated its application potential in these fields and has begun to flourish. However, few studies have reviewed the application of MOFs in the management of fluorine-containing gases [75–80]. A comprehensive analysis of the adsorption mechanism at the molecular level is crucial for developing high-performance porous adsorbents [1, 40, 81].

In this review, we thoroughly reviewed the adsorption and separation of F-gases by MOFs in various application scenarios. The works on balancing the adsorption capacity and selectivity toward different F-gases by modifying the pore morphology, size, interacting sites, and framework flexibility of MOFs are summarized. Emphasis was placed on mechanisms for separating highly similar fluorine-containing gas components. We aimed to provide a reference for future MOFs design by classifying and analyzing molecular interaction between F-gases and MOFs and interpreting detailed mechanisms behind their high performance, instead of simply repeating existing work in chronological order or making a league table of the selectivity and adsorption capacities of the designs. In addition, we discussed challenges faced in the further development of MOF adsorbents for purifying F-gases for industrial applications and proposed future research opportunities and prospects.

## Adsorption and Separation of PFCs

### PFCs Capture from PFCs/N_2_ Mixtures

**Fig. 2:**
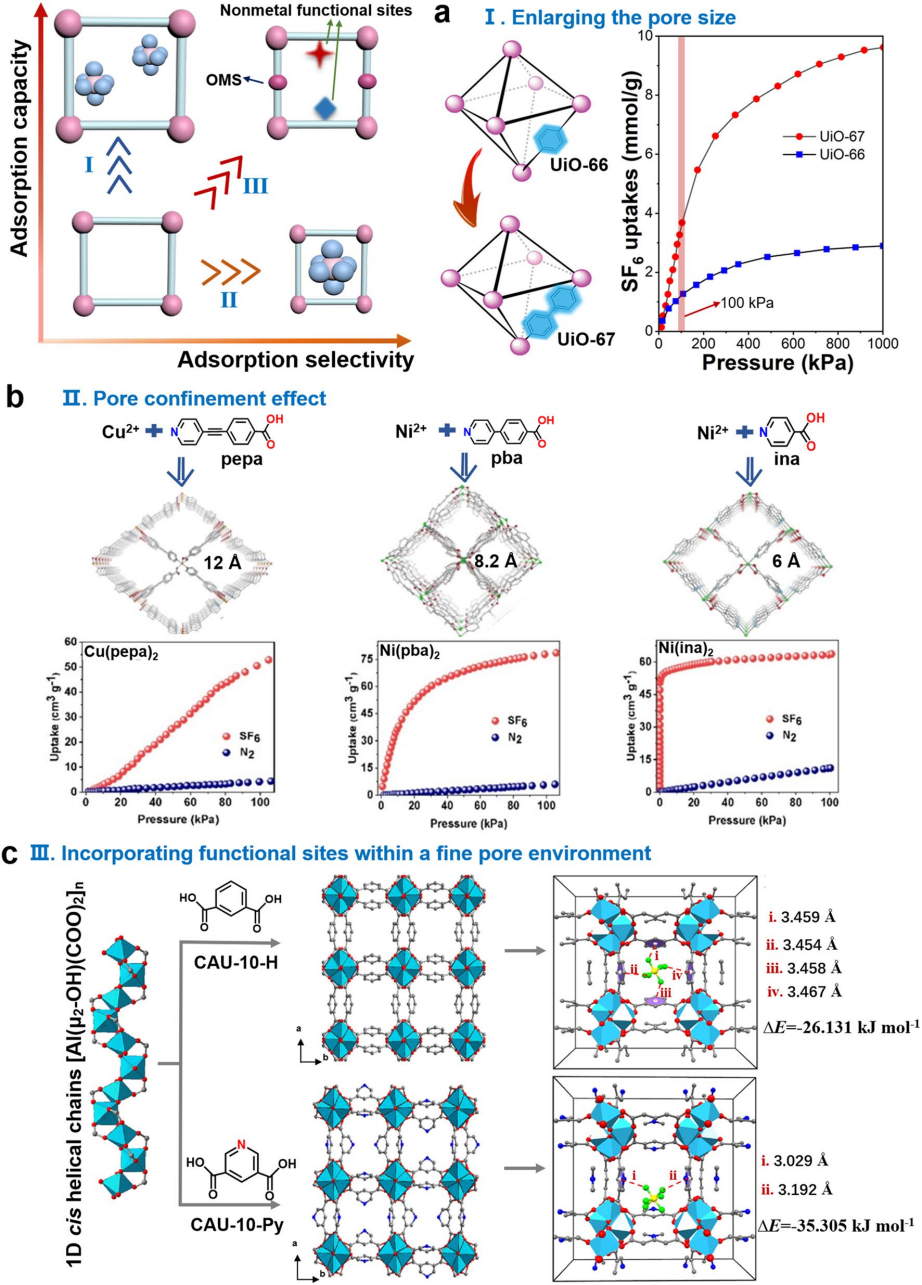
Three strategies for improving PFCs adsorption/separation by MOFs: (I) enlargement of pore size improves adsorption capacity for PFCs; (II) pore size constraint improves the adsorption selectivity of PFCs/N_2_; and (III) the introduction of metal or nonmetal sites breaks the trade-off between adsorption selectivity and adsorption capacity. **a** Adsorption isotherms of SF_6_ on UiO-66 and UiO-67 at 298 K. **b** Pore structure and adsorption isotherms of Cu(peba)_2_, Ni(pba)_2_, and Ni(ina)_2_. **c** Crystal structures of CAU-10-H and CAU-10-Py and binding sites with SF_6_

PFCs, mainly including SF_6_, CF_4_, NF_3_, and C_3_F_8_, are irreplaceable gases in the semiconductor industry and often used as plasma-etching and plasma-cleaning gases. Most PFCs remain intact (60%–70% of the original component) in the manufacturing process. Given their high GWP and extremely long atmospheric lifetime (Table 1), used PFCs usually need to be removed through combustion and thermal plasma decomposition [1, 82]. However, these methods can not only recover F-gases but also produce inorganic fluorine waste, which needs further treatment [17, 43, 83]. An alternative solution to reduce PFCs consumption in the production process is to mix low-content PFCs with inert gases (typically N_2_). This approach is effective because a high-concentration N_2_ fluid mixture (with SF_6_/N_2_ volume ratios often set to 0.1, 0.01, 0.002, or 0.0003) maintains a dielectric strength comparable to that of pure PFCs [84–91]. To meet the demand for high purity in power industry (SF_6_ > 99%) and other industries, realize the recycling of expensive resources and reduce the environmental burden, the development of an effective physical adsorption process separating and recycling PFCs in PFCs/N_2_ mixtures is highly desired, which require sorbents with ideal adsorption selectivity and capacity for PFCs even at low concentrations. MOFs are potential sorbents for PFCs separation because of their highly designable pore chemistry. In this section, we will discuss satisfactory performance of MOFs for capturing PFCs from PFCs/N_2_ mixtures in terms of adsorption capacity, selectivity, and the trade-off between capacity and selectivity through pore environment and host–guest interaction regulation (Fig. 2). Current researches on PFCs adsorption by MOFs are at the initial stage and mostly focus on SF_6_ capture (Table 2).

**Table 1:** Properties of typical PFCs and N_2_

Gases [Refs.]	Molecular configurations and size	Kinetic diameter (Å)	Boiling points (K)	GWP (CO_2_ = 1)	Atmospheric lifetime (years)	Polarizability (× 10^–25^ cm^−3^)	Dipole moment (× 10^–18^ esu‧cm)
SF_6_ [87, 92, 93]		5.2	209.25	22800	3200	65.4	0
CF_4_ [86, 94]		4.8	145.11	7390	50,000	38.4	0
NF_3_ [22, 23]		4.5	144.11	17,200	740	36.2	0.235
C_3_F_6_ [95–97]		~ 6.0	236.3	100	10	63.5	–
C_3_F_8_ [98–100]		~ 6.0	242	7000	> 2600	68.0	–
N_2_ [88, 101]		3.8	77.35	–	–	17.4	0

**Table 2:** Properties, chemical compositions, and structures of various MOFs for adsorption and separation of PFCs mixtures (PFCs/N_2_ and PFCs/PFCs)

F-gases	MOFs [Refs.]	Structures and Chemical compositions	Channel size (Å)	BET surface area (m^2^ g^−1^)	IAST	Adsorption amount (mmol g^−1^)	Thermodynamic stability	Kinetic stability	Chemical stability	Main interactions
SF_6_	Cu(peba)_2_ [92, 155]	Cu(NO_3_)_2_·3H_2_O, 4-(pyridin-4-ylethynyl)benzoic acid (peba)	12	814.3	SF_6_/N_2_ = 18.2^a^ (v/v = 0.1)	2.36^a^	Stability up to 300 °C	Stable after 10 cycles		Pore confinement effect; vdW
	Ni(pba)_2_ [92, 156]	Ni(NO_3_)_2_·6H_2_O, 4-(4-pyridyl)benzoic acid	8.2	807.2	SF_6_/N_2_ = 200.6^a^ (v/v = 0.1)	3.50^a^	Stability up to 400 °C	Stable after 10 cycles		Pore confinement effect; vdW
	Ni(3-mpba)_2_ [126]	Ni(NO_3_)_2_·6H_2_O, 3-methyl-4-(pyridin-4-yl)benzoic acid	11.93	835.1	SF_6_/N_2_ = 221^a^ (v/v = 0.1)	2.83 ^a^	Stability up to 380 °C	Stable after 5 cycles		Functional group; vdW
	Ni(ina)_2_ [92, 157]	Ni(CH_3_COO)_2_·4H_2_O, isonicotinic acid	6	470	SF_6_/N_2_ = 375.1^a^ (v/v = 0.1)	2.84^a^, 2.39^b^	Framework decomposition occurs after 400 °C	Stable after 10 cycles		Pore confinement effect; vdW
	Ni(adc)(dabco)_0.5_ [112]	NiCl_2_⋅6H_2_O, H_2_adc, 1,4-benzenedicarboxylate (bdc) and 1,4-diazabicyclo[2.2.2]octane (dabco)	5.1	743.9	SF_6_/N_2_ = 948.2^a^ (v/v = 0.1)	2.38^a^, 2.23^b^	Brilliant thermal stability	Stable after 10 cycles	Excellent resistance to water vapor	Pore confinement effect; vdW (F…π)
	Cu-MOF-NH_2_ [123]	Cu(NO_3_)_2_·3H_2_O, NH_2_-H_4_tptc	6, 10, 13	2145	SF_6_/N_2_ = 266^a^ (v/v = 0.1)	7.88^a^, 3.39^b^	Stability up to 270 °C	Stable after 10 cycles	Unstable in water environments	vdW (N − H…F)
	SBMOF-1 [106, 107]	CaCl_2_, 4,4′-sulfonyldibenzoic acid (H_2_sdb)	5 − 8.5	169.33	SF_6_/N_2_ = 727^a^ (v/v = 0.0003), 325^a^ (v/v = 0.1)	1.02^a^		Stable after 5 cycles	Good structural stability	Aromatic functionalized channels; noncovalent interaction enhance the pore confinement effect
	CAU-10-H [101]	Al_2_(SO_4_)_3_⋅18H_2_O, m-Na_2_bdc	5.2	684.4	SF_6_/N_2_ = 122.6 (v/v = 0.1)	1.00^a^; 1.07^a^ (273 K); 0.68^b^	Stability up to 400 °C			Pore size; functional group
	CAU-10-Py [101]	AlCl_3_⋅6H_2_O, 3,5-pyridinedicarboxylic acid (pydc)	5.2	935.9	SF_6_/N_2_ = 203.6 (v/v = 0.1)	1.76^a^; 2.11^a^ (273 K); 1.13^b^	Stability up to 420 °C	Stable after 6 cycles		Pore size; functional group
	Co-MOF (GNU-3) [125]	Co(NO_3_)_2_⋅6H_2_O, H_2_ipa	5.5 × 5.5 Å^2^	930.08	SF_6_/N_2_ = 317.6^a^ (v/v = 0.1), 133.63^a^ (v/v = 0.01)	2.63^a^	Stability up to 370 °C	Stable after 5 cycles	Good water stability	Pore size; vdW
	M-MOF-74 (M = Mg, Co, Zn) [118]	Metallic salt, benzenedicarboxylate (dobdc)	11	992 − 1631	SF_6_/N_2_ = 18.2^a^ − 46^a^ (v/v = 0.1)	3.71–6.42^a^				OMS
	HBU-21 [116]	In(NO_3_)_3_⋅4H_2_O, 9-(4-carboxyphenyl)−9H-carbazole-3,6-dicarboxylic acid	6.14	381.44	SF_6_/N_2_ = 184.05^a^ (v/v = 0.1)	0.949^a^	Stability up to 400 °C	Recyclable and reusable	Stable in acetone, but prone to collapse in distilled water and anhydrous methanol	OMS
	CTH-18 [158]	MnCl_2_∙4H_2_O, 1,2,3,4,5,6-Hexakis(4-carboxyphenyl)benzene (H_6_cpb)	4.3, 5.1	354	SF_6_/N_2_ = 29^a^ (v/v = 0.1)	~ 1.9^a^, ~ 1.55^b^	Stable before 425 °C		Stable in organic solvents	Pore confinement effect; vdW
	HKUST-1 (Cu-BTC or MOF-199) [1, 14, 109, 121, 131]	Cu(NO_3_)_2_·3H_2_O, 1,3,5-benzene tricarboxylic acid (H_3_btc)	7 ~ 12	1428 − 1880	SF_6_/N_2_ = 127^a^ (v/v = 0.1)	6.71^a^	Stable before 370 °C	Minor decrease after 7 cycles	Structural decomposition occurs in water or humid air	OMS
	Im_1%_@HKUST-1 [121]	Cu(NO_3_)_2_·3H_2_O, H_3_btc, imidazole	6.4	1211.0	SF_6_/N_2_ = 93^a^ (v/v = 0.1)	5.98^a^	Stable before 370 °C	Minor decrease after 7 cycles	Great water stability	Functional groups; OMS
	MIL-100 [93, 159, 160]	FeCl_3_ solutions, btc	25, 29	1772	24.4^a^	2.95^a^	Stable before 270 °C			OMS
	MIL-100 granule [161]	Fe(NO_3_)_3_·9H_2_O, btc	-	1619	SF_6_/N_2_ = 24.4^a^ (v/v = 0.1)	1.658^a^		Stable after 5 cycles	Great water stability	OMS
	MIL-101 [93, 119, 162]	Cr(NO_3_)_3_·9H_2_O, terephthalic acid	29, 34	2162.01	SF_6_/N_2_ ~ 24^a^	2.01^a^				Pore size; OMS
	Co_2_(1,4-bdc)_2_(dabco) [93, 163]	Co(NO_3_)_2_·6H_2_O, bdc, dabco	7.6	1600		3.39^a^	Stable before 500 °C			Framework flexibility
	MOFF-5 [111]	Cu(NO_3_)_2_·2.5H_2_O, fluorinated tetrazole	22	2445		1.74	Framework decomposition occurs at approximately 220 °C		Poor stability in water and under humid conditions	Fluorine functional groups
	UiO-66 [102, 127, 142]	ZrCl_4_, bdc	8, 11	1074	SF_6_/N_2_ = 74^a^ (v/v = 0.1), 130^a^ (v/v = 0.002)	1.45^a^ (293 K)	Good thermal stability		Good chemical, mechanical stability	vdW
	UiO-66-Br_2_ [127]	ZrCl_4_, bdc-Br		616	SF_6_/N_2_ = 200^a^ (v/v = 0.1)	0.92^a^	Good thermal stability	Stable after 20 cycles	Good chemical and mechanical stability	Pore confinement effect; polar functional groups enhance vdW effect
	UiO-67[105]	ZrCl_4_, biphenyl-4,40-dicarboxylate (H_2_bpdc)	12, 23	2411	SF_6_/N_2_ = 37 (v/v = 1, 10 bar), 30 (v/v = 0.1, 10 bar)	~ 3.7^a^, 9.66 (10 bar)		Stable after 20 cycles		Pore size
	Yb-TBAPy [164]	Yb(C_2_H_3_O_2_)_3_ 4H_2_O, 1,3,6,8-tetrakis(4-carboxyphenyl)pyrene(H_4_TBAPy)	6.4 − 6.9	940	SF_6_/N_2_ = 47^a^ (v/v = 0.1, 293 K)	2.33^a^ (293 K)	Stability up to 400 °C		Stable under conditions other than acidic (1 M HCl, pH = 1) and alkaline (1 M NaOH, pH = 14) environments	Pore confinement effect; vdW
	Tm-TBAPy [164]	Tm(C_2_H_3_O_2_)_3_ xH_2_O, H_4_TBAPy	6.4 − 6.9	716	SF_6_/N_2_ = 48^a^ (v/v = 0.1, 293 K)	1.83^a^ (293 K)	Stability up to 400 °C		Stable under conditions other than acidic (1 M HCl, pH = 1) and alkaline (1 M NaOH, pH = 14) environments	Pore confinement effect; vdW
	M_3_(HCOO)_6_ (M = Co, Ni, Mn) [83]	Co(NO_3_)_2_·6H_2_O, formic acid	5 –6	330.1	SF_6_/N_2_ = 120^a^ (v/v = 0.01)	2.17^a^ (Co)				Polar functional groups (charge exchange between the F atom in F-gases and the H atom in HCOO^−^); pore confinement effect
	BUT-53 [124]	Co(OAc)_2_·4H_2_O, H_2_dpb	4 –8	866	SF_6_/N_2_ = 2485^a^ (v/v = 0.1)	3.55^a^, 2.82^b^	Stability up to 550 °C		Superior water stability	F…H interaction
CF_4_	Ni(adc)(dabco)_0.5_ [86, 112]	NiCl_2_⋅6H_2_O, H_2_adc, dabco	5.1	743.9	CF_4_/N_2_ = 23^a^ (v/v = 0.1)	1.76^a^, 0.52^b^	Brilliant thermal stability	Stable after 5 cycles	Excellent resistance to water vapor	Pore confinement effect; vdW (F…π)
	Ni(ina)_2_ [23]	Ni(CH_3_COO)_2_·4H_2_O, Isonicotinic acid	6	434	CF_4_/N_2_ = 34.7^a^ (v/v = 0.1)	2.92^a^, 1.15^b^	Stable before 400 °C			vdW
	NH_2_-Ni-MOF (Ni(3-ain)_2_) [23]	Ni(NO_3_)_2_·6H_2_O, 3-aminoisonicotinic acid	5	872	CF_4_/N_2_ = 46.3^a^ (v/v = 0.1)	2.69^a^, 1.25^b^	Stable before 400 °C			Amino functional groups
	JUCXEK [22]	actinide metal Np and organic ligand oxalic acid	6.26	1253.1	CF_4_/NF_3_ = 50.15^a^ (v/v = 0.05)	0.8 (20 bar, v/v = 0.05)		Stable after 12 cycles		Pore confinement effect; vdW; electrostatic interaction
	Zn(fba) [113]	C_4_H_10_O_6_Zn, benzoic acid	5	345	CF_4_/NF_3_ = 29^a^ (v/v = 0.76)	~ 1.05^a^	Stable before 500 °C		Good water stability	vdW; close match with the arene-lined pores
	LIFM-90 [138]	ZrCl_4_, bpdc	6.96 –7.97	2933.4	CF_4_/CH_4_ = 4.6	2.0				OMS; pore size
	Co_2_(1,4-bdc)_2_(dabco) [93, 163]	Co(NO_3_)_2_·6H_2_O, bdc, dabco	7.6	1600		0.714^a^	Stable before 500 °C			Framework flexibility
	MIL-100 [93, 159, 160]	FeCl_3_ solutions, btc	25, 29	1772		0.53^a^	Stable before 270 °C			OMS
	MIL-101 [93, 119, 162]	Cr(NO_3_)_3_·9H_2_O, terephthalic acid	29, 34	2162.01		0.53^a^				Pore size; OMS
	MOFF-5 [111]	Cu(NO_3_)_2_·2.5H_2_O, fluorinated tetrazole	22	2445		0.09	Framework decomposition occurs at approximately 220 °C		Poor stability in water and under humid conditions	Fluorine functional groups
	UiO-66 [102, 127, 142]	ZrCl_4_, bdc	8, 11	1074		~ 0.75^a^	Good thermal stability		Good chemical and echanical stability	vdW
	UiO-66-Br_2_ [127]	ZrCl_4_, bdc-Br	9	616		~ 0.68^a^	Good thermal stability	Stable after 20 cycles	Good chemical and mechanical stability	Pore confinement effect; polar functional groups enhance vdW effect
	SBMOF-1 [107]	CaCl_2_, sdb	5 − 8.5	169.33	CF_4_/N_4_ = 18.21(v/v = 0.01, (*P/P*_0_ = 1))	1.01 (*P/P*_0_ = 1)			Good structural stability	Aromatic functionalized channels; noncovalent interaction enhance the pore confinement effect
	M_3_(HCOO)_6_ (M = Co, Ni, Mn) [83]	Co(NO_3_)_2_·6H_2_O, formic acid	5 − 6	330.1	CF_4_/N_2_ = 11.6^a^ (v/v = 0.01)	1.42^a^				Polar functional groups (charge exchange between the F atom in F-gases and the H atom in HCOO^−^); pore confinement effect
NF_3_	Ni(ina)_2_ [23]	Ni(CH_3_COO)_2_·4H_2_O, isonicotinic acid	6	434	NF_3_/N_2_ = 35.8^a^ (v/v = 0.1)	2.69^a^	Stable before 400 °C			vdW
	NH_2_-Ni-MOF (Ni(3-ain)_2_) [23]	Ni(NO_3_)_2_·6H_2_O, 3-aminoisonicotinic acid	5	872	NF_3_/N_2_ = 32.4^a^ (v/v = 0.1)	2.62^a^	Stable before 400 °C			Amino functional groups
	SBMOF-1 [107]	CaCl_2_, sdb	5–8.5	169.33		1.17 (*P/P*_0_ = 1)			Good structural stability	Aromatic functionalized channels; noncovalent interaction enhance the pore confinement effect
	M_3_(HCOO)_6_ (M = Co, Ni, Mn) [83]	Co(NO_3_)_2_·6H_2_O, formic acid	5–6	330.1	NF_3_/N_2_ = 19.2^a^ (v/v = 0.01)	1.46^a^				Polar functional groups (charge exchange between the F atom in F-gases and the H atom in HCOO^−^); pore confinement effect
BF_3_	M-MOF-74 (M = Mg, Co, Zn) [14]	Metallic salt, dobdc	11	992–1631		0.9 bar: 7.3 (Co-MOF-74), 3.5 (Mn-MOF-74), 2.1 (Mg-MOF-74)		Stable after 10 cycles		OMS
C_3_F_8_	Co_x_Cr-MIL-101 [119]	Cr(NO_3_)_3_·9H_2_O, Co(NO_3_)_2_⋅6H_2_O, terephthalic acid	14 –20, 22−30	2162.01–2518.14	C_3_F_8_/N_2_ = 146.7^a^	0.078 (180 Pa)	Good thermal stability	Good cyclic stability		OMS; unique layered porous structure
C_3_F_6_	Ca-tcpb [142]	CaCl_2_, 1,2,4,5-tetrakis(4-carboxyphenyl) benzene (H_4_tcpb)		260.65	C_3_F_6_/C_3_F_8_ > 10000^a^ (v/v = 0.01)	2.0^a^	Stable up to approximately 400 °C	Stable after 5 cycles		Kinetic sieving; vdW
C_6_F_14_	MOFF-5 [111]	Cu(NO_3_)_2_·2.5H_2_O, fluorinated tetrazole	22	2445		6.65	Framework decomposition occurs at approximately 220 °C		Poor stability in water and under humid conditions	Fluorine functional groups
	Compound 2 [128]	ZrCl_4_, tpdc-(CF_3_)_2_	11, 16	2819		142 wt% (50 Torr)	Stable up to approximately 200 °C	Stable after 10 cycles	Great water stability	Fluorine functional groups

298 K. ^a^ At 1 bar. ^b^ At 0.1 bar

Void ratio and pore size are key factors for PFCs adsorption in porous materials, especially when capture capacity is considered. An early study on SF_6_ adsorption in UiO-66 (pore window size: 8, 11 Å) and MIL-100 (pore window size: 25 Å) demonstrated that MIL-100 with higher Brunauer–Emmett–Teller (BET) surface area (1947 m^2^ g^−1^ for MIL-100, 1333 m^2^ g^−1^ for UiO-66) adsorbed a higher amount of SF_6_ (2.59 mmol g^−1^) than UiO-66 (1.45 mmol g^−1^) at 293 K and 1 bar, suggesting that PFCs adsorption capacity can be increased by using MOFs with large void ratios and pore size [102]. Compared with traditional porous materials, such as activated carbon, highly porous MOFs have well-developed reticular chemistry and are thus easy to design [84, 103, 104]. For example, when a long linker (biphenyl-4,4′-dicarboxylate) was replaced with benzene-1,4-dicarboxylic acid in UiO-66 for the preparation of UiO-67 (Fig. 2a), the larger pore size (12 and 23 Å) and higher BET surface area (2411 m^2^ g^−1^) in UiO-67 led to a higher SF_6_ adsorption capacity (3.7 mmol g^−1^) [105]. However, UiO-66 (~ 37) had higher SF_6_/N_2_ selectivity predicted by ideal adsorption solution theory (IAST) at 298 K and 1 bar than UiO-67 (~ 21). These results suggest that only increasing the pore size of MOFs may lead to a trade-off between adsorption capacity and selectivity.

Strong interactions between PFCs and MOFs are conducive to adsorption selectivity for PFCs/N_2_ separation. Regulating the pore size of MOFs to be close to that of PFCs can enhance host–guest van der Waals (vdW) interaction through the pore confinement effect and enhance adsorption affinity. For example, SBMOF-1 has a similar pore size (6.703 × 7.840 Å) to SF_6_ (6.13 × 6.13 × 6.13 Å), which can achieve an IAST selectivity of up to 325 for SF_6_/N_2_ (*v*/*v* = 0.1) at 298 K and 1 bar [106, 107]. The appropriate pore size in SBMOF-1 contributes to the strong hydrogen bonding interactions between hydrogen atoms on the benzene ring, and the fluorine atoms of SF_6_. Ni(ina)_2_ (pore size: 6 Å), Ni(pba)_2_ (8.2 Å), and Cu(peba)_2_ (12 Å) with similar diamond networks were synthesized using pyridine carboxylic acid ligands with different length [92]. Ni(ina)_2_ with the closest kinetic diameter to SF_6_ (5.2 Å) has a selectivity of as high as 375.1 (SF_6_/N_2_, *v*/*v* = 0.1 at 298 K and 1 bar) (Fig. 2b), and its high isosteric heat of adsorption (the *Q*_st_ for Ni(ina)_2,_ Ni(pba)_2_, and Cu(peba)_2_ are 33.4, 24.0 and 14.4 kJ mol^−1^, respectively) further confirms affinity (Fig. 2c). Density functional theory (DFT) calculation and Grand canonical Monte Carlo (GCMC) simulation reveals the reason from the molecular point of view, calculated using the forcite module with a universal force field (UFF) for the geometrically optimized single-crystal frameworks of these three MOFs, and also employing a *Q*_qe_ fitted charges [108]. The pore structure of Ni(ina)_2_ is perfectly matched with SF_6_, resulting in a significantly shorter interaction distance (2.74 − 3.18 Å) between the fluorine atom of SF_6_ and the H atom on the pyridine ring of MOF than that of Ni(pba)_2_ (3.06 − 4.26 Å) and Cu(peba)_2_ (3.11 − 3.27 Å). The SF_6_ interaction sites are highly consistent with the results revealed by single-crystal X-ray diffraction (SCXRD). And the molecular simulation shows that the binding energies (∆*E*) of SF_6_ with Ni(ina)_2_, Ni(pba)_2_, and Cu(peba)_2_ are 59.08, 38.21, and 30.18 kJ mol^−1^, respectively, which also aligns with the trend of *Q*_st_ values obtained from experiments. In addition, the adsorption capacity of Ni(ina)_2_ for SF_6_ at 0.1 bar (2.39 mmol g^−1^) is higher than that of most reported MOFs (Table 2), such as HKUST-1 (1.37 mmol g^−1^) [109], HKUST-1c (1.32 mmol g^−1^) [91], UU-200 (0.99 mmol g^−1^) [110], DUT-9 (0.80 mmol g^−1^) [93], Zn_4_O(dmcpz)_3_ (1.12 mmol g^−1^) [93], and MOFF-5 (0.09 mmol g^−1^) [111], further demonstrating its potential to capture SF_6_ at a low partial pressure. Both Ni(ina)_2_ (IAST = 34.7, 298 K, 1 bar) and Ni(adc)(dabco)_0.5_ (IAST = 23, 298 K, 1 bar) exhibit good selectivity for CF_4_/N_2_, but achieving industrial application requires good water stability [23, 86, 112]. The MOF structure Zn(fba) synthesized from fluorine-containing aromatic ligands not only possesses the water stability inherent in Zn-MOFs, but also shows excellent equilibrium selectivity for CF_4_ (the IAST of CF_4_/N_2_ is 29 at 298 K and 1 bar) due to the close matching of small spherical pores (5 Å) within its corrugated channel, which is the highest selectivity reported for CF_4_ in a water-stabilized MOFs [113]. However, MOFs with pore size comparable to that of PFCs often do not exhibit remarkable adsorption capacities due to the limited pore volume.

Both adsorption capacity and selectivity are crucial to PFCs capture and separation performance. Introducing interaction sites into MOF’s apertures allow for extraordinary host–guest interactions for PFCs sorption might be the key to balancing selectivity and adsorption capacity. MOFs with open metal sites (OMS) usually have strong electrostatic interactions between metal sites and F-gases with certain polarity or charge distribution [114–117]. The OMS-containing series of isostructured M-MOF-74 (*M* = Co, Mg, Zn) exhibit remarkable adsorption capacities for SF_6_ at 298 K and 1 bar, and Mg-MOF-74 (BET surface area = 1631 m^2^ g^−1^) and Co-MOF-74 (BET surface area = 1219 m^2^ g^−1^) reach as high as 6.42 and 5.34 mmol g^−1^, respectively [118]. The adsorption capacity of Zn-MOF-74 (BET surface area = 992 m^2^ g^−1^) under the same conditions is relatively lower (3.79 mmol g^−1^) but still higher than the adsorption capacities of MOFs with comparable specific surface area but no OMS (such as 2.36 mmol g^−1^ for Cu(peba)_2_; Table 2), and the selective adsorption capacity of SF_6_/N_2_ is as high as 46. The introduction of accessible cobalt ions into MIL-101’s framework enriches its active sites and simultaneously improves the adsorption capacity of Co_0.2_Cr-MIL-101 for C_3_F_8_ (0.078 mmol g^−1^ at 298 K and 1.8 bar) and the selectivity of C_3_F_8_/N_2_ (IAST = 146.7 at 298 K and 1 bar), which are 3.03 and 14.3 times the adsorption capacity and selectivity of the original MIL-101, respectively [119]. However, OMS in MOFs usually preferentially coordinate with water, causing the competitive adsorption of H_2_O and low water stability of MOFs when moisture is present in the gas mixture. For example, the OMS-containing HKUST-1 has an excellent adsorption capacity for SF_6_ (6.71 mmol g^−1^, 298 K, and 1 bar), but its structure irreversibly decomposes once exposed to water or humid air, which significantly limits its application [120]. However, after the introduction of imidazole (Im) ligand into HKUST-1, water stability was significantly enhanced due to the pre-attached of Im to the unsaturated Cu sites [121, 122]. There was also no significant decrease in Im-HKUST-1 performance after several adsorption/desorption cycles. Doping MOFs with hybridized ligands is an effective strategy to enhance their adsorption and water stability properties.

Given that PFCs have multiple fluorine atoms in their molecular structures, introducing hydrogen bonding receptors into a pore surface might enhance the affinity of MOFs with PFCs. The hydrogen atom on the –NH_2_ group introduced into Cu-MOF-NH_2_ can simultaneously form hydrogen bonds with the three fluorine atoms (N−H…F) of SF_6_, and cooperates with vdW interactions and pore confinement effect afforded to the framework by the calix[3]zrene-analogous nanospace, enabling the selective adsorption of SF_6_ from mixtures with N_2_ [123]. At 298 K and 1 bar, the benchmark adsorption of SF_6_ reaches 7.88 mmol g^−1^, with an IAST selectivity of 266 (v/v = 0.1). The Co(II)-pyrazolate MOF (BUT-53) exhibits excellent SF_6_ adsorption capacity (3.55 mmol g^−1^) and unprecedented SF_6_/N_2_ selective adsorption (IAST = 2485, v/v = 0.1) at 1 bar and 298 K, and can efficiently recover high-purity (> 99.9%) SF_6_ from low-concentration (10%) mixtures through breakthrough experiments [124]. These features are due to F…H interactions between the benzene and pyrazole groups of dipyrazolate (dpb) ligands on BUT-53 and SF_6_, and dpb ligands afford MOFs dynamic molecular traps that can accommodate guest molecules of different sizes. The charge exchange between H atoms from formic acid and F atoms from PFCs (SF_6_, CF_4_ and NF_3_) in M_3_(HCOO)_6_ (M=Co, Ni, Mn) channel results in PFC adsorption performance exceeding that of N_2_, and the restriction of appropriate pore size on gas molecules further enhances the affinity between host and guest [83]. At 298 K and 1 bar, the adsorption capacities of Co_3_(HCOO)_6_ are 2.17 mmol g^−1^ for SF_6_ and only 0.16 mmol g^−1^ for N_2_, which exhibits outstanding SF_6_/N_2_ selective adsorption (v/v = 0.01 for SF_6_/N_2_, IAST = 120). A Co-MOF (GNU-3) containing a H_2_ipa ligand has a localized nanomolecular trap, which offsets the trade-off effect of SF_6_ using functional pore surface modification and suitable pore size [125]. The adsorption position of SF_6_ in GNU-3 was visualized by GCMC simulation (the frame and gases used the *Q*_eq_ method for atomic partial charges and the UFF adopted by Lennard–Jones parameters) and combined with DFT calculations to reveal the adsorption behavior within the MOFs. Through strong hydrogen bonding interactions with the benzene ring of the framework and the hydrogen atoms of the ipa^2−^ in the pore, SF_6_ is trapped in the polyaromatic ring-functionalized channel centers and nanomolecular traps in the pore walls. At 298 K and 1 bar experimental conditions, activated GNU-3a exhibits an SF_6_ selectivity of 317.6 (SF_6_/N_2_, v/v = 0.1) with an adsorption capacity of 2.63 mmol g^−1^, which closely aligns with the adsorption trends and capacities (about 2.75 mmol g^−1^) obtained from theoretical calculations. In addition, GNU-3 has excellent thermal/chemical stability and kinetic stability, and its cost-effective raw materials and environmentally friendly synthesis method highlight its potential for industrial applications.

Compared with Ni(pba)_2_, Ni(3-mpba)_2_ obtained by introducing methyl into pores has more binding sites with SF_6_ and smaller pores, which enhances affinity with SF_6_, and thus shows improved adsorption selectivity for SF_6_/N_2_ (IAST = 221 at 298 K and 1 bar, v/v = 0.1) and adsorption capacity (2.83 mmol g^−1^) [126]. As shown in Fig. 2c, CAU-10-Py obtained by replacing the benzene ring (CAU-10-H) with a pyridine ring not only increases the adsorption selectivity from 122.6 to 203.6 (298 K, 1 bar, v/v = 0.1) but also doubles the adsorption capacity under low- (0.1 bar) and high-pressure (1 bar) conditions [101]. The reasons for the improved were deeply analyzed from a molecular perspective by using GCMC simulations (DREIDING force field for nonmetallic atoms, UFF force field for Al) to understand the interactions between SF_6_ (S and F, with characteristic energies (*Ɛ*) of 165.14 and 27.02 K, collision diameters (*δ*) of 3.228 and 2.947 Å, and charges (*q*) of 0.660 and − 0.110 *e*, respectively) and MOFs, NVT-Monte Carlo (NVT-MC) simulations to determine the preferential adsorption sites of the adsorbates within the MOFs frameworks, and DFT calculations to ascertain the optimal adsorption configurations and bonding energies. The introduction of nitrogen atoms alters the electrostatic potential distribution in the pore, which leads to the adsorption site of SF_6_ moving to the edge of the channel. The interaction between the pyridine ring and SF_6_ (the distance is 3.029–3.192 Å, ∆*E* = − 35.305 kJ mol^−1^) is stronger than the S–F…π interaction in CAU-10-H (the distance is 3.454–3.467 Å, ∆*E* = − 26.131 kJ mol^−1^), and the pore diameter is enlarged by the substitution of the benzene ring by pyridine (CAU-10-Py is 5.9 Å and CAU-10-H is 5.7 Å), which enables pores to accommodate SF_6_. The overall trend of adsorption behavior between simulation and experiment is consistent. Given that the polarizability of absorbing atoms is closely related to vdW force, introducing highly polarizable functional groups into MOFs can enhance vdW interactions with PFCs and thus improves the selective adsorption of PFCs/N_2_. The adsorption of SF_6_ and CF_4_ was investigated after the introduction of different polar functional groups (–NH_2_, –NO_2_, –Cl, –Br, –Br_2_, –I) into UiO-66 [127]. The results show that UiO-66-Br_2_, characterized by high polarization, considerably enhances the uptake of SF_6_ and CF_4_ under industrially relevant low-pressure conditions (298 K, < 0.05 bar). Moreover, UiO-66-Br_2_ has high SF_6_/N_2_ selectivity at low (IAST = 560–580, v/v = 0.0003) and high concentrations (v/v = 0.1, IAST = 200) and can exhibit superior performance in separating SF_6_/N_2_ to UiO-66 under dynamic mixed flow conditions. Under the guidance of reticular chemistry, three isostructural MOFs with varying linker lengths and fluorine contents were synthesized from Zr-MOFs, utilizing fluorinated phenyl or biphenyl linkers [128]. Among them, compounds 2 and 3 not only exhibit high water stability and thermal stability, but can also absorb over 140 wt% of C_6_F_14_ within a few minutes at 300 °C and a partial pressure of 50 Torr. This indicates that the functionalization of the framework structure with trifluoromethyl groups contributes to enhancing the hydrophobicity of the MOFs, thereby further improving its adsorption capacity.

Both adsorption capacity and selectivity are crucial indicators for the adsorption and separation of PFCs by MOFs, and are the primary considerations for the development of new MOFs adsorbents. At present, the research subject of adsorptive separation of PFCs/N_2_ is still robust MOFs. The adsorption capacity for PFCs can be effectively enhanced by modulating the void ratio and pore size of MOFs, but the results suggest that increasing the pore size alone may lead to a trade-off between adsorption capacity and selectivity. Controlling the pore size to be close to the diameter of the PFCs can enhance the adsorption selectivity through pore confinement effect, but the limited pore size tends not to exhibit excellent adsorption capacity. To overcome this trade-off, researchers have combined the introduction of metal/nonmetal sites to further regulate the pore environment, which is currently an important method to achieve efficient adsorption and separation of PFCs/N_2_ by MOFs.

### Separation of Gas Mixtures of Different PFCs Species

Apart from separating PFCs/N_2_ mixtures, separating different species of PFCs gas mixtures is of great importance to the electronic industry. The physical and chemical properties of PFCs and N_2_ are quite different, especially in polarity, facilitating separation. However, the separation of different PFCs species is extremely difficult due to the highly similar physicochemical properties, requiring MOFs with high molecular recognition capability. To date, few studies have explored the separation of different PFCs species by MOFs.

In a synthetic route, CF_4_ is the impurity with the highest content in NF_3_ products, seriously affecting the etching of NF_3_ in semiconductor chip production [24, 129–131]. For the identification of suitable MOF candidates for CF_4_/NF_3_ separation, JUCXEK (JUCXEK is a structured crystalline substance combining actinide Np with the organic ligand oxalic acid [132, 133]) was screened from 796 kinds of MOFs in the CoRe MOF database through high-throughput GCMC calculations (metal elements used the UFF force field [134], while nonmetal elements used the DREIDING force field [135]) with a selectivity of up to 50.15 (298 K, 1 bar, *v*/*v* = 0.05/0.95) for CF_4_/NF_3_ [22]_._ To better understand the relationship between properties and guest structures, unlike previous studies that considered the NF_3_ and CF_4_ models are only single spheres with zero charge and zero natural electrostatic interaction energy, the four- and five-site models of NF_3_ (N: *δ* = 4.28 Å, *Ɛ* = 173.86 K, *q* = 0.084 *e*) and CF_4_ (C: *δ* = 4.66 Å, *Ɛ* = 134 K, *q* = 0.76 *e*) were established by adding virtual atoms F_N (*q* = − 0.028 e) and F_C (*q* = − 0.19 *e*), respectively [136, 137]. The performance is attributed to the relative enhancement of vdW interactions because the pore-limiting diameter (6.26 Å) of JUCXEK is close to the CF_4_ molecule (4.66 Å), and the charge of each atom of CF_4_ is higher than that of NF_3_ leading to enhances electrostatic interactions between CF_4_ and JUCXEK. When the metal position is optimized, Zr-JUCXEK shows industrial value in simulated pressure swing adsorption (298 K, 1 bar, v/v = 0.05/0.95), with both CF_4_ outlet concentration (< 20 ppm) and NF_3_ recovery (88.9%). This work demonstrates the potential application of computational chemistry in the separation of fluorine compounds [89, 90, 138–140].

The formation of C_2_F_6_ in an industrial process is mainly catalyzed by fluoroethanes, such as CF_3_CH_2_F and CF_3_CHF_2_ with metal fluoride or fluorine gas, at high temperatures, resulting in a mixture of residual CF_3_CH_2_F or CF_3_CHF_2_ in C_2_F_6_. Ni(pba)_2_ with fourfold intermingled structure exhibits high selectivity to CF_3_CH_2_F/C_2_F_6_ (v/v = 5/95, IAST = 38.8) and CF_3_CHF_2_/C_2_F_6_ (v/v = 5/95, IAST = 19.8) at 298 K and 1 bar [141]. The breakthrough experiment shows that this approach can directly obtain high-purity C_2_F_6_ from the ternary mixture of CF_3_CH_2_F/CF_3_CHF_2_/C_2_F_6_ (v/v/v = 5/5/90) with a yield of 7.1 mol kg^−1^. In addition to the C–H…F interactions between three F-gases and Ni(pba)_2_, the C–H bond in CF_3_CH_2_F and CF_3_CHF_2_ can enhance the interaction with pore surface through additional C–H…π interactions, thus allowing for the one-step purification of C_2_F_6_ in CF_3_CH_2_F/CF_3_CHF_2_/C_2_F_6_ mixtures.

**Fig. 3:**
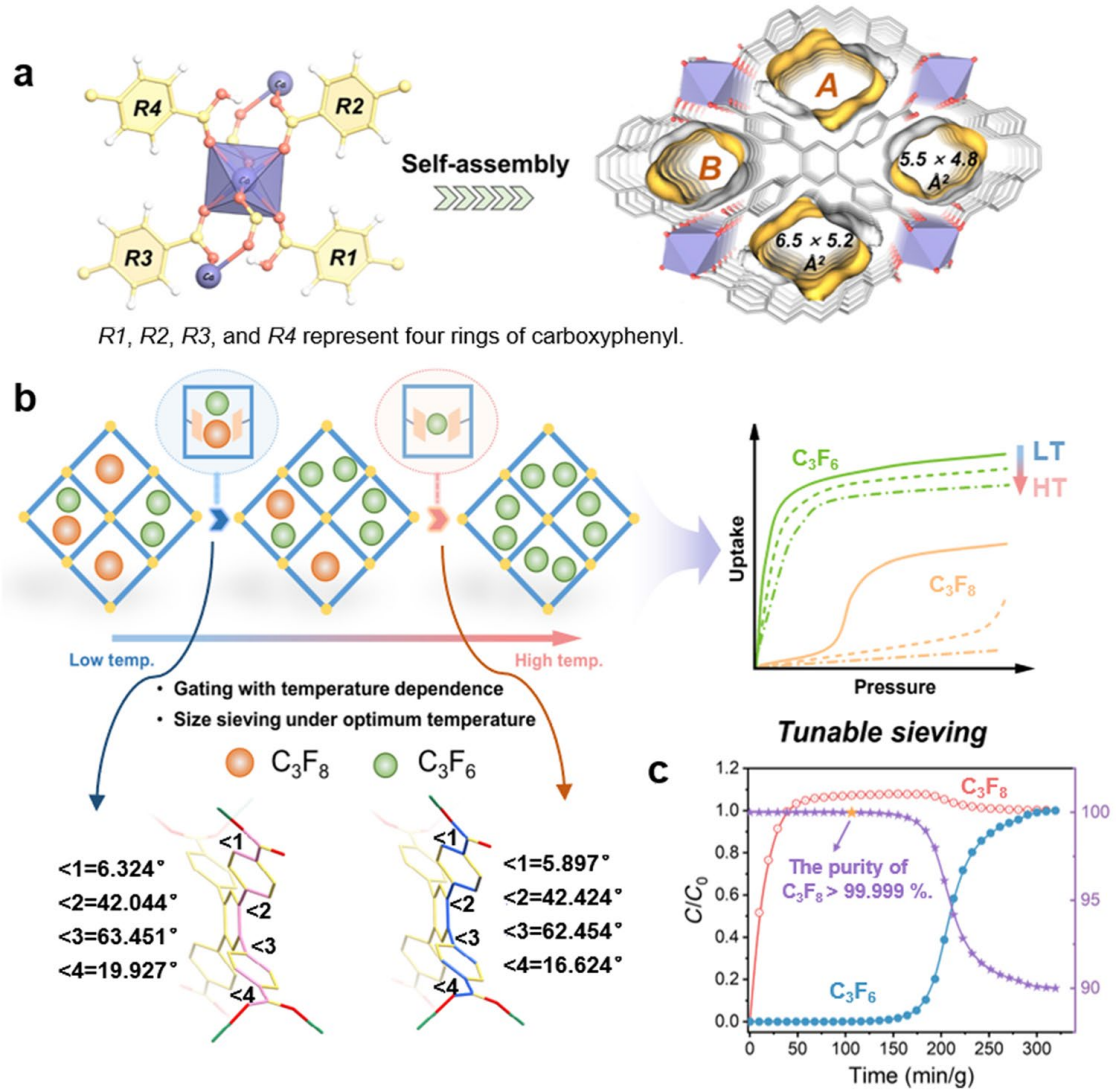
**a** Local coordination environment of H_2_tcpb^2−^ with Ca (Ca is purple, C is yellow, O is rose red, and H is white) and the single-crystal structure of Ca-tcpb (A and B represent two types of one-dimensional channels). **b** Schematic of adjustable molecular sieve for promoting C_3_F_8_/C_3_F_6_ mixture by a thermal regulation–gating effect, difference before and after H_2_tcpb^2−^ adsorption frame C_3_F_6_, and the adsorption isotherms of C_3_F_8_ and C_3_F_6_ at different temperatures. **c** Breakthrough curve of Ca-tcpb on the packed column bed of a mixture of C_3_F_6_/C_3_F_8_ (v/v = 0.1) at 298 K and 1 bar

A typical method for producing C_3_F_8_ involves the direct fluorination of C_3_F_6_ but leads to 1%–10% residual C_3_F_6_ in products, whereas high-purity C_3_F_8_ (more than 99.999%) is usually used in various industries [98, 143]. Thus, further purifying C_3_F_8_/C_3_F_6_ with similar physical and chemical properties is important. The adsorption separation of C_3_F_8_/C_3_F_6_ is considered to be one of the most difficult challenges in the field of adsorbents, because the subtle physicochemical differences between C_3_F_8_ and C_3_F_6_ (Table 1) need to be realized through the ultraprecise modulation of adsorbents [144]. At present, the researches mainly based on robust MOFs for F-gases capture and separation, but the adsorption–desorption kinetics and absorption in confined space are usually low, limiting the pore editing of rigid frames at the sub-angstrom level [145–147]. Flexible MOFs refer to a novel type of porous materials, which can undergo dynamic structural changes (“breathing” or “swelling” behavior) in response to external stimuli (temperature, pressure, light, or molecular exchanges of the object), which is theoretically useful for the separation of gas mixtures [54, 148–151]. However, flexible MOFs often exhibit low thermal and chemical stability, and will co-adsorb other gases with the “gate-open” process caused by a certain gas, which are not suitable for gas separation [152]. Flexibility–rigidity can keep its whole structure under low pressure and show its significant plateau. At the same time, the flexible-robust MOFs structure usually show an adsorbate-dependent gas sorption isotherm, which can be used for the separation of different gas mixtures [153, 154]. An adsorbent with flexible–robust microporous was reported, Ca-tcpb, which used the influence of temperature on the varying degrees of the local twisting and vibrational motion of benzene ring ligands (R1, R2, R3, R4) to adjust the gating effect, thus reducing the co-adsorption of the C_3_F_6_/C_3_F_8_ mixture (Fig. 3a, b). At different temperatures, Ca-tcpb remains porous for small C_3_F_6_, but its gating pressure increases with temperature for the larger C_3_F_8_, enabling the efficient sieving of C_3_F_6_/C_3_F_8_ at ambient pressure (1 bar) by simply modulating the temperatures. (The IAST reaches 10,000 at 298 K.) And its adsorption performance on C_3_F_6_ was almost unchanged after 20 adsorption/desorption cycles. The dynamic penetration experiment (Fig. 3c) of C_3_F_6_/C_3_F_8_ (v/v = 0.1) shows that Ca-tcpb can preferentially adsorb C_3_F_6_ at 106.5 min g^−1^ (298 K), resulting in a high-purity C_3_F_8_ (99.999%) [142, 145–147]. This work demonstrates the unique advantages of flexible–robust MOFs in separating highly similar PFCs.

For the adsorptive separation of PFCs/PFCs with highly similar structures, editing the pores of robust MOFs at the sub-angstrom level becomes exceedingly challenging. Flexible–robust MOFs, with unique flexible structure, enable precise tuning of the pore environment while overcoming the problems of poor structural stability and co-adsorption of flexible MOFs. They can maintain structural integrity at low pressure and exhibit adsorbate-dependent gas sorption isotherms, making them more suitable for the molecular screening of complex separation systems. Flexible–robust MOFs have shown great potential in the emerging field of F-gases adsorption and separation, warranting further study.

## Adsorption and Separation of CFCs, HCFCs, and HFCs

In the 1930s, Thomas Midgely, Albert L. Henne, and Robert R. McNary discovered fluorocarbon refrigerants. CFCs and HCFCs refrigerants were widely used as second-generation refrigerants [143, 165]. Due to the serious consumption of ozone layer by CFCs, the Montreal Protocol in 1987 signed a restriction order about the production of ozone-depleting substances, such as CFCs. The Copenhagen Amendment in 1922 called for the gradual phaseout of HCFCs by 2030 [166, 167]. Over the past few decades, the market has been dominated by third-generation refrigerants, called HFCs, but some of them have been banned or are being phased out due to their high GPW characteristics [40]. Table 3 lists the physical properties of several typical common fluorocarbon refrigerants. To control the emission of these gases, MOFs have been developed to capture fluorocarbon refrigerants that have azeotropic or near-azeotropic characteristics and are difficult to separate through conventional low-temperature distillation (Table 4). Their recovery through MOFs can be performed for the production of fourth-generation refrigerant and can prevent the direct release of refrigerants to the atmosphere.

**Table 3:** Physical properties of common fluorocarbon refrigerants

Species (R-number) [Refs.]	Chemical formula	Kinetic Diameter (Å)	Molecular Weight	Boiling Point (K)	Dipole Moment (D)	Polarizability (× 10^–25^ cm^−3^)	GWP (CO_2_ = 1)
CFC-12 [81, 111, 168]	CCl_2_F_2_	4.4	120.91	243.5	0.51	63.7	10,890
HCFC-22 [169–173]	CHClF_2_	4.2	86.46	232.1	1.46	44.7	5280
HFC-32 [168, 174, 175]	CH_2_F_2_	3.9	50.02	221.5	1.98	26.5	675
HFC-125 [16, 176, 177]	CHF_2_CF_3_	4.4	120.02	224.9	1.56	43.6	3500
HFC-134a [77, 178–180]	CH_2_FCF_3_	–	102.03	246.9	2.06	43.2	1430
HFC-143a [40, 80, 169]	CH_3_CF_3_	–	84.04	225.8	2.34	40.4	4470

**Table 4:** Properties, structures and compositions of various MOFs for adsorption/separation of fluorocarbon refrigerants

F-gases	MOFs [Refs.]	Structures and Chemical compositions	Channel size (Å)	BET surface area (m^2^ g^−1^)	IAST	Adsorption amount (mmol g^−1^)	Thermodynamic stability	kinetic stability	chemical stability	Main interactions
R12	M-MOF-74 (M = Ni, Co) [168, 196]	Ni(NO_3_)_2_·6H_2_O or Co(NO_3_)_2_·6H_2_O, dobdc	11	940 − 1135	–	4.58 (*P*/*P*_0_ = 0.01)				OMS
	MIL-100(Fe) [160]	FeCl_3_ solutions, btc or trimesic acid	5–8, 24 –30	~ 2000	–	6.7 (4 bar)				OMS
	MIL-101(Fe) [168]	FeCl_3_·6H_2_O, bdc	11.7, 16, 25–29	4230	–	15 (4 bar), < 2 (*P*/*P*_0_ = 0.01)				OMS (C–F_R12_…Cr^3+^)
	MOFF-5 [111]	Cu(NO_3_)_2_·2.5H_2_O, fluorinated tetrazole	22	2445		6.6	Framework decomposition occurs at approximately 220 °C		Poor stability in water and under humid conditions	Fluorine functional groups. vdW
R32	MIL-101(Fe) [168]	FeCl_3_·6H_2_O, bdc	11.7, 16, 25−29	4230	–	8 (2 bar)				OMS
	UiO-66 [172]	ZrCl_4_, bdc	8, 11	1383		2.9^a^	Good thermal stability	Over 15 adsorption–regeneration cycles still stable		Defect sites; vdW
	HKUST-1 (Cu-BTC or MOF-199) [174, 197]	Cu(NO_3_)_2_·3H_2_O, H_3_btc	7–12	1172	303.15 K, R32/R125 (v/v = 70/30) = 0.091^b^, 1.91 (6 bar), 4.29 (10 bar)	303.15 K: 0.921 (0.09 bar), 8.58^a^, 13.1 (13.13 bar)	Stability up to 350 °C		The host backbone will be damaged in humid air	OMS
	ZIF-8 [174, 198]	Zn(NO_3_)_2_·6H_2_O, 2-methylimidazole	12 –17	1302	303.15 K, R32/R125 (v/v = 70/30) = 0.238^b^, 0.652 (6 bar), 0.897 (10 bar)	303.15 K: 0.0856 (0.09 bar), 1.53 (1 bar), 8.17 (13.13 bar)	High thermal stability		Great water stability	vdW; ligand rotation
	MOF-177 [174, 199]	Zn(NO_3_)_2_·6H_2_O, benzene-1,3,5-tribenzoic acid (H_3_btb)	17–20, 20–25	4190	303.15 K, R32/R125 (v/v = 70/30) = 0.355^b^, 0.557 (6 bar), 0.741 (10 bar)	303.15 K: 0.295 (0.23 bar), 1.64 (1.21 bar), 30 (12.99 bar)				vdW
	MIL-53 [174, 200]	Al(NO_3_)_3_·9H_2_O, bdc	8 –10	1043	303.15 K, R32/R125 (v/v = 70/30) = 0.113^b^, 0.801 (6 bar), 1.15 (10 bar)	303.15 K: 0.437 (0.07 bar), 3.12 (1.14 bar), 6.71 (11.57 bar)				vdW
R22	DUT-67 [169, 201]	ZrCl_4_, 2,5-thiophenedicarboxylate (tdc)	5.8, 11.2	1300	R22/CO_2_ = 51.4–33.3^b^ (v/v = 10/90, 50/50, 90/10)	5.54^a^, 6.21 (6.23 bar)	Stability up to 220 °C	The performance of the six adsorption/desorption cycles is stable	Having good water stability and stability in extreme environments such as NaOH (1 M), HCl (6 and 12 M) and boiling water	Cl/F… H − O and O…H − C hydrogen bonds
	LIFM-66 [170]	ZrCl_4,_ H_3_btb, 4′,4‴,4⁗′,4″′′′′-(ethene-1,1,2,2-tetrayl)tetrakis ([1,1′-biphenyl] −4-carboxylate) (H_4_ettc)	16.2	3631	R22/N_2_ = 186^a^ (v/v = 1/99)	9.8^a^	Stability up to 450 °C		Great stability in water and harsh chemical environments (pH = 2 − 10)	Strong O…H − C_FC_ and O − H…F_FC_ interactions, weak vdW forces
	LIFM-67 [170]	ZrCl_4,_ = 5ˈ-(4-carboxyphenyl)−2′,4′,6′ -trimethyl-[1,1′,3′,1″-terphenyl]−4,4″-dicarboxylic acid (H_3_ctta), H_4_ettc	15.5	2904	R22/N_2_ = 154^a^ (v/v = 1/99)	11.2^a^	Stability up to 450 °C		Great stability in water and harsh chemical environments (pH = 2 − 10)	Methyl functional group (strong O…H − C_FC_ interactions, weak C − H…F_FC_ and C − H…Cl_FC_ van der Waals forces)
	LIFM-100 [194]	Cu(NO_3_)_2_ 2.5HO, 2,2′-bis(trifluomethyl)[1,1′-biphenyl]−4,4′-dicarboxylate acid	14.3	663	R22/N_2_ = 399.1	3.1 (273 K, 1 atm), 1.6 (1 atm)	Stability up to 250 °C		High chemical stability	vdW (interaction between fluorine atoms and R22)
	LIFM-26 [171]	Fe_3_O(H_2_O)_3_, 2,3,5,6-tetrachloride terephthalic acid (H_2_tcdc)	12.6	1513	R22/N_2_ = 202 (1 atm, v/v = 10/90)	6.5^a^	Stability up to 200 °C		Great water and chemical (pH = 1, 7, 11.5) stability	OMS; functional polar groups
	LIFM-28 [192]	ZrCl_4,_ 2,2’-bis (trifluoromethyl)−4,4’-biphenyldicarboxylate	11 − 13	940	R22/N_2_ = 42 (v/v = 10/90, 273 K)	~ 2.29^a^	Good thermal stability		Stable in boiling water and harsh environments (pH = 1, 12)	Functional groups; pore space partition
	UiO-66 [172]	ZrCl_4_, bdc	8, 11	1383		5^a^	Good thermal stability	Over 15 adsorption–regeneration cycles still stable		Defect sites; vdW
	PCN-700 [173, 202]	ZrCl_4_, 2,2'-dimethylbiphenyl-4,4'-dicarboxylic acid (H_2_Me_2_-bpdc)	14.3	879	R22/N_2_ ~ 123^a^ (v/v = 10/90, 273 K)	~ 2.9^a^	Stability up to 450 °C			vdW
	LIFM-90 [173]	C_101_H_73_N_3_O_32_Zr_6_ PCN-700, 2,2'-dimethylbiphenyl-4,4'-dicarboxylic acid (H_2_L^1^), 2'-(azidomethyl)-[1,1':4',1'' terphenyl]−4,4''-dicarboxylic acid (H_2_L^2^)	13.2	2222	R22/N_2_ ~ 270^a^ (v/v = 10/90, 273 K)	~ 8.43^a^	Stability up to 450 °C			Functional groups
	LIFM-91 [173]	LIFM-90, CuI, propargylamine	13.2	1674	R22/N_2_ ~ 342^a^ (v/v = 10/90, 273 K)	~ 7.1^a^	Stability up to 450 °C			Functional groups; pore space partition
	LIFM-92 [173]	PCN-700, H_2_L^1^, 2',5'-bis(azidomethyl)-[1,1':4',1''terphenyl]−4,4''-dicarboxylic acid (H_2_L^3^)	13.2	2175	R22/N_2_ ~ 400^a^ (v/v = 10/90, 273 K)	~ 7.89^a^	Stability up to 450 °C	Stable after 3 cycles		Functional groups
	LIFM-93 [173]	LIFM-92, CuI, propargylamine	12.7	1577	R22/N_2_ ~ 223^a^ (v/v = 10/90, 273 K)	~ 5.94^a^	Stability up to 450 °C	Stable after 3 cycles		Functional groups; pore space partition
	LIFM-94 [173]	PCN-700, H_2_L^1^, 2',3',5',6'-tetrakis(azidomethyl) [1,1':4',1''-terphenyl]−4,4''-dicarboxylic acid (H_2_L^4^)	13.2	1657	R22/N_2_ ~ 308^a^ (v/v = 10/90, 273 K)	~ 6.31^a^	Stability up to 450 °C			Functional groups
	LIFM-95 [173]	LIFM-94, CuI, propargylamine	12.7	1496	R22/N_2_ ~ 231^a^ (v/v = 10/90, 273 K)	~ 6.50^a^	Stability up to 450 °C			Functional groups; pore space partition
	MAF-X10 [181]	Zn(NO_3_)_2_·6H_2_O, bdc, 3,3',5,5'-tetramethyl-4,4'-bipyrazole (H_2_bpz)	9.4 × 9.9 × 13.2 Å^3^	2032		~ 9.48^a^	Stability up to 550 °C	The adsorption–desorption at 313 K can reach equilibrium within 50 s		Pore size and shape; C_R22_…N, C_R22_…O
	MAF-X12 [181]	Zn(NO_3_)_2_·6H_2_O, H_2_bpz, 1,4-dicarboxylic acid (H_2_ndc)	9.4 × 9.9 × 13.2 Å^3^	1787		~ 8.1^a^	Stability up to 450 °C	The adsorption–desorption at 313 K can reach equilibrium within 50 s		Pore size and shape; C_R22_…N, C_R22_…O
	MAF-X13 [181]	Zn(NO_3_)_2_·6H_2_O, H_2_bpz, biphenyl-4,4'-dicarboxylic acid (H_2_bpdc)	9.4 × 9.9 × 15.9 Å^3^	2742		11.22^a^	Stability up to 450 °C	The adsorption–desorption at 313 K can reach equilibrium within 50 s		Pore size and shape; C_R22_…N, C_R22_…O
	MIL-101(Fe) [168]	FeCl_3_·6H_2_O, bdc	11.7, 16, 25 − 29	4230	-	9.83^a^; 11 (2 bar)				OMS; C–H…π/C–H…O
R125	UiO-66 [172, 176, 177]	ZrCl_4_, bdc	8, 11	1383		4.6^a^	Good thermal stability	Over 15 adsorption–regeneration cycles still stable		Defect sites; vdW
	HKUST-1 [174, 176, 197]	Cu(NO_3_)_2_·3H_2_O, H_3_btc	7 –12	1172	303.15 K, R32/R125/R134a (v/v/v = 47/21/32): R125/R32 = 9.05^b^, 0.644 (4 bar), 0.422 (6 bar)	303.15 K: 2.39 (0.05 bar), 5.74^b^, 6.85 (11.14 bar)	Stability up to 350 °C		The host backbone will be damaged in humid air	OMS
	ZIF-8 [174, 176, 198]	Zn(NO_3_)_2_·6H_2_O, 2-methylimidazole	12–17	1302	303.15 K, R32/R125/R134a (v/v/v = 47/21/32): R125/R32 = 4.18^b^, 1.58 (4 bar), 1.28 (6 bar)	303.15 K: 0.307 (0.05 bar), 3.23^a^, 4.51 (11.14 bar)	High thermal stability		High water stability	vdW; ligand rotation
	MOF-177 [174, 199]	Zn(NO_3_)_2_·6H_2_O, H_3_btb	17– 20, 20 − 25	4190	303.15 K, R32/R125/R134a (v/v/v = 47/21/32): R125/R32 = 2.9^b^, 1.79 (4 bar), 1.46 (6 bar)	303.15 K: 0.437 (0.13 bar), 4.11 (0.99 bar), 17.8 (11.16 bar)				vdW
	MIL-53 [174, 200]	Al(NO_3_)_3_·9H_2_O, bdc	8 –10	1043	303.15 K, R32/R125/R134a (v/v/v = 47/21/32): R125/R32 = 6.91^b^, 1.46 (4 bar), 1.18 (6 bar)	303.15 K: 0.757 (0.04 bar), 2.54 (1.10 bar), 3.75 (9.61 bar)				vdW
R134a	DUT-67 [169, 201]	ZrCl_4_, tdc	5.8, 11.2	1300	R134a/CO_2_ = 31.1 − 25.8 (0.01 bar, v/v = 10/90, 50/50, 90/10)	5.18^a^, 6.12 (6.23 bar)	Stability up to 220 °C	The performance of the 6 adsorption/ desorption cycles is stable	Having good water stability and stability in extreme environments such as NaOH (1 M), HCl (6 M and 12 M) and boiling water	Cl/F…H − O and O…H − C hydrogen bonds
	MOFF-5 [111]	Cu(NO_3_)_2_·2.5H_2_O, fluorinated tetrazole	22	2445		5.4	Framework decomposition occurs at approximately 220 °C		Poor stability in water and under humid conditions	Fluorine functional groups
	LIFM-66 [170]	ZrCl_4_ H_3_btb, H_4_ettc	16.2	3631	R134a/N_2_ = 232^a^ (v/v = 1/99)	10.6^a^	Stability up to 450 °C		Great stability in water and harsh chemical environments (pH = 2 ~ 10)	Strong C − H…F_FCF_ interactions and weak vdw forces
	LIFM-67 [170]	ZrCl_4_ H_3_ctta, H_4_ettc	15.5	2904	R134a/N_2_ = 154^a^ (v/v = 1/99)	11.1^a^	Stability up to 450 °C		Great stability in water and harsh chemical environments (pH = 2 ~ 10)	Methyl functional group (strong C − H…F_FCF_ interactions, weak O…H − C_FCF_ and C − H…F_FCF_ vdW forces)
	Ni-MOF-74 [178, 182]	Ni(CH_3_COO)_2_·4H_2_O or Ni(NO_3_)_2_·6H_2_O, dobdc	11	1150	–	4.7 (0.12 bar), about 4.98 (6 bar)	Framework decomposition occurs after 400 °C			CH_2_F moiety of R134a interacts with the Ni^2+^
	Ni-MOF-74-BPP [178, 182]	Ni(CH_3_COO)_2_·4H_2_O or Ni(NO_3_)_2_·6H_2_O, biphenyl with *para*-COOH (bpp)	17	2040	–	3.63 (0.12 bar), aabout 7.37 (6 bar)	Framework decomposition occurs after 400 °C			Pore size; OMS
	Ni-MOF-74-TPP [178, 179, 182]	Ni(CH_3_COO)_2_·4H_2_O or Ni(NO_3_)_2_·6H_2_O, triphenyl with *para*-COOH (tpp)	23	1980	-	2.74 (0.12 bar), ~ 7.63 (6 bar)	Framework decomposition occurs after 400 °C	Stable performance after more than 50 cycles		Pore size; OMS
	NU-1000 [179, 203]	ZrOCl_2_·8H_2_O benzoic acid, H_4_TBAPy	12, 30	2259	–	17 (~ 6 bar)	Framework decomposition occurs after 400 °C	The performance of the 20 adsorption/desorption cycles is stable		vdW
	PCN-222 [179, 204]	ZrOCl_2_·8H_2_O, benzoic acid, FeTCPP	11, 30	1869	-	13 (~ 6 bar)	Stability up to 500 °C			vdW
	MIL-101(Cr) [179, 205]	Cr(NO_3_)_3_·9H_2_O, H_2_bdc	11, 16, 23 − 29	2642	–	~ 14.3 (~ 6 bar)	Stability can reach about 380 °C			vdW; OMS
	Ni_3_(C_2_O_4_)_3_(tpt)_2_ (MCF-61) [180]	NiCl_2_, dimethyl oxalate, 2,4,6-tris(4-pyridyl)−1,3,5-triazine (tpt)	20	2096	–	11.2 (5.34 bar), 10.78 (4.88 bar)	Stability up to 300 °C			Pore size
	Ni_3_(C_2_O_4_)_3_(tppa)_2_ (MCF-62) [180]	NiCl_2_, dimethyl oxalate, tris(4-(4-pyridyl)-phenyl)amine (tppa)	33	2630	–	17.03 (5.28 bar), 16.6 (4.88 bar)	Stability up to 300 °C			Pore size
	Ni_3_(C_2_O_4_)_3_(tppt)_2_ (MCF-63) [180]	NiCl_2_, dimethyl oxalate, 2,4,6-tris(4-(4-pyridyl)-phenyl)−1,3,5-triazine (tppt)	37	2749	–	20.29 (5.72 bar), 18.6 (4.88 bar)	Stability up to 300 °C			Pore size
	HKUST-1 [174, 197]	Cu(NO_3_)_2_·3H_2_O, H_3_btc	7–12	1172	303.15 K, R32/R125/R134a (v/v/v = 47/21/32): R134a/R32 = 8.71^b^, 1.76 (4 bar), 1.33 (6 bar); R134a/R125 = 0.962^b^, 2.73 (4 bar), 3.15 (6 bar)	303.15 K: 2.91 (0.04 bar), 6.62 (0.98 bar), 7.35 (5.27 bar)	Stability up to 350 °C		The host backbone will be damaged in humid air	OMS
	ZIF-8 [174, 198]	Zn(NO_3_)_2_·6H_2_O, 2-methylimidazole	12–17	1302	303.15 K, R32/R125/R134a (v/v/v = 47/21/32): R134a/R32 = 7.63^b^, 2.96 (4 bar), 2.47 (6 bar); R134a/R125 = 1.83^b^, 1.87 (4 bar), 1.93 (6 bar)	303.15 K: 0.194 (0.04 bar), 3.87 (0.98 bar), 4.85 (5.27 bar)	High thermal stability		High water stability	vdW; ligand rotation
	MOF-177 [174, 199]	Zn(NO_3_)_2_·6H_2_O, H_3_btb	17–20, 20–25	4190	303.15 K, R32/R125/R134a (v/v/v = 47/21/32): R134a/R32 = 3.88^b^, 3.48 (4 bar), 2.98 (6 bar); R134a/R125 = 1.34^b^, 1.94 (4 bar), 2.04 (6 bar)	303.15 K: 0.534 (0.11 bar), 13.5 (0.92 bar), 19.3 (4.7 bar)				vdW
	MIL-53[174, 200]	Al(NO_3_)_3_·9H_2_O, bdc	8–10	1043	303.15 K, R32/R125/R134a (v/v/v = 47/21/32): R134a/R32 = 12.0^b^, 1.96 (4 bar), 1.50 (6 bar); R134a/R125 = 1.73^b^, 1.35 (4 bar), 1.27 (6 bar)	303.15 K: 0.40 (0.01 bar), 2.49 (1.11 bar), 3.34 (4.71 bar)				vdW
R13 (CFC-13, CFCl_3_)	MIL-101(Fe) [168]	FeCl_3_·6H_2_O, bdc	11.7, 16, 25 − 29	4230	–	4 (2 bar)				OMS
R113 (CFC-113, CFCl_2_CF_2_Cl)	MOFF-5 [111]	Cu(NO_3_)_2_·2.5H_2_O, fluorinated tetrazole	22	2445		10.09^a^	Framework decomposition occurs at approximately 220 °C		Poor stability in water and under humid conditions	Fluorine functional groups

298 K. ^a^ At 1 bar. ^b^ At 0.1 bar. The single-crystal structure of PCN-700 and LIFM-90–95 are derived from Ref. [173]. Copyright 2017, Royal Society of Chemistry

### Adsorption of Fluorocarbon Refrigerants

**Fig. 4:**
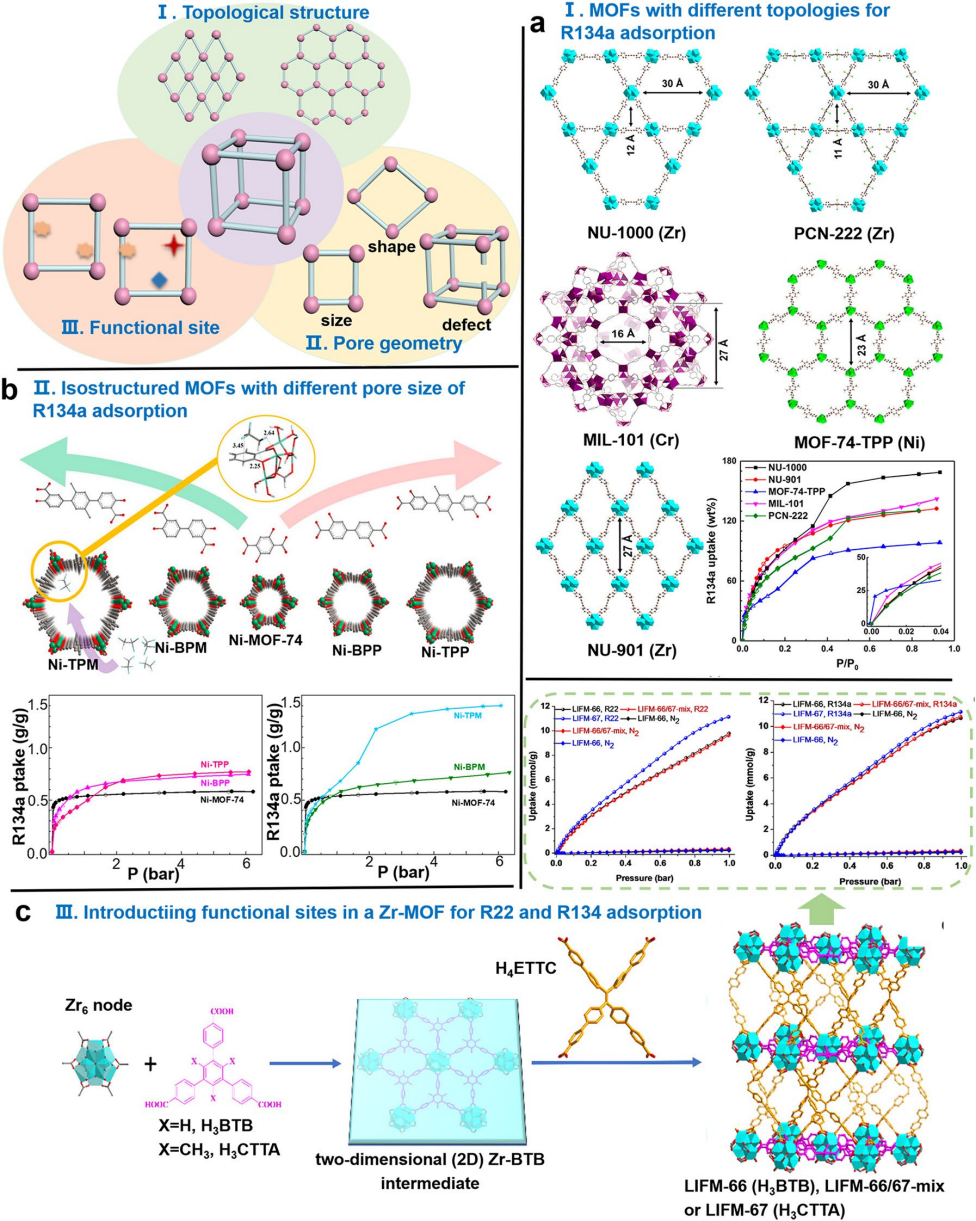
Topological structure (I), pore geometry (II), and functional sites (III) that affect the adsorption of fluorocarbon refrigerants by MOFs. **a** Single-crystal structure of five representative MOFs with different topological structures and isothermal adsorption curve of R134a at 298 K. **b** Structural features of pore-engineered Ni-MOF-74 members and the isothermal adsorption curve of R134a at 298 K. **c** Synthesis procedure, single-crystal structure, and the isothermal adsorption (298 K) of R22 and R134a of LIFM-66, LIFM-66/67-mix, and LIFM-67

The diverse topological structures arising from different metal nodes and coordination can directly alter the pore structure of MOFs, thereby affecting the accessibility of fluorocarbon refrigerants (Fig. 4). By exploring a series of mesoporous MOFs with different topological structures but the same chemical properties, it was found that two MOFs with similar pore size show excellent adsorption capacities for R134a, namely, NU-1000 composed of mesoporous hexagonal (~ 30 Å) and microporous triangular channels (~ 12 Å) and MIL-101 connected by microporous pentagonal (~ 11 Å) and hexagonal windows (~ 16 Å; Fig. 4a) [179]. However, NU-1000 shows an S-shaped isotherm because of its two-dimensional extended Kagome network structure, which enhances saturation capacity by approximately 170 wt%. Meanwhile, MIL-101 only produces 60% working capacity with I-shaped isotherm. This analysis reveals that a structure with micropore and mesopore combinations has higher absorption performance than an ordered adsorbent with one shape and uniform pore size. This unique composition of micropores and macropores permits the stepwise adsorption isotherm showing strong host–guest interaction as well as sorbate–sorbate interactions with sufficient pore volume. These properties may be the reason that the micro- and mesoporous MOF-177 is superior to other microporous MOFs (Cu-BTC, MIL-53(Al), and ZIF-8) for HFCs (R134a, R32, and R125) adsorption [174]. MOF-177 has adsorption rates more than twofold those of the other three microporous materials. Therefore, MOFs with hierarchical micro/mesoporous structure may be suitable for the capture of fluorocarbon refrigerants in terms of adsorption capacity.

Without changing the topological structure, finely adjusting pore size and shape by changing ligands or introducing pore defects directly affects the gas adsorption performance of MOFs. A study on the adsorption behavior of R22 in the presence of isoreticular MOFs (MAF-X10, MAF-X12, and MAF-X13) showed that the subtle modification of the pore size and shape of sorbents induced by the length and side groups of ligands can dramatically improve adsorption performance [181]. MAF-X13 with the largest pore size allows for the saturable uptake of R22 (11.22 mmol g^−1^) at 293 K and 1 bar and is higher than that of MAF-X10 and MAF-X12. Ni-MOF-74 members, Ni-MOF-74 (11 Å), Ni-BPP (17 Å), Ni-BPM (19 Å), Ni-TPP (23 Å), and Ni-TPM (27 Å) are constructed by adjusting the relative locations of hydroxyl and carboxyl groups in dihydroxyterephthalic acid ligand phenyl ring analogs (Fig. 4b), with the largest pore sizes of Ni-TPM showing a considerably high saturation capacity for R134a, especially under high-pressure conditions, exhibiting a 300% increase in saturation capacity compared with Ni-MOF-74 (the saturation absorption of Ni-TPM is higher than 13.7 mmol g^−1^) [178, 182]. The initial adsorption of R134a occurs near nickel nodes, and it interacts with the inorganic nodes and organic linkers of Ni-TPM to form a monolayer as pressure increases. Pore filling occurs at high pressure. By contrast, pore filling in Ni-MOF-74 and Ni-BPP with small pore size usually occurs at low pressure. The adsorption of R12 by microporous M-MOF-74 (M = Ni, Co) and mesoporous MIL-101 shows the same trend, and the adsorption capacity of M-MOF-74 (4.58 mmol g^−1^) is more than twice that of MIL-101 (< 2 mmol g^−1^) under low-pressure conditions (*P/P*_0_ = 0.01) and saturates at *P*/*P*_0_ = 0.05, while the saturation capacity of MIL-101 (15 mmol g^−1^) is twice that of M-MOF-74 at 4 bar (*P/P*_0_ = 0.6) [168]. Under the condition of the same ligand bridging length, the effective pore size of isotactic mesoporous MOFs (MCF-61, MCF-62, and MCF-63) with unique honeycomb-like structure is fourfold the ligand bridging length (5.5, 8.8, and 9.9 Å). Thus, pore size can be easily increased and fine-tuned, and resulting changes directly affect the adsorption performance of R134a [180]. The saturated adsorption of MCF-63 for R134a (20.29 mmol g^−1^) with the highest pore volume (2.36 cm^2^ g^−1^) and BET surface area (2749 m^2^ g^−1^) is almost the same as that of activated carbon Maxsorb III, which has a surface area of over 3000 m^2^ g^−1^. Its isotherm shape is related to its pore size and exhibits excellent adsorption thermal conversion performance. Introducing defect sites into MOFs is also an effective method for generating additional voids or pores and adjusting gas adsorption isotherms [183, 184]. In the capture experiments of R125 by UiO-66 (6–7 Å), Cu-BTC (5.5, 10, and 12 Å), and ZIF-8 (11 Å), UiO-66, which has low pore size and surface area, shows the exceptional adsorption capacity (7 mmol g^−1^) because of the presence of defects in its structure, which facilitate the formation of additional pores and accessible adsorption sites [16, 174, 176, 177, 185].

Introducing nonmetallic/metallic functional sites for the regulation of the pore environment can enhance the affinity of MOFs for fluorocarbon refrigerants. Isostructural MOFs (LIFM-66, LIFM-66/67-mix, and LIFM-67) can be prepared by introducing methyl groups that modulate the adsorption performance of R22 and R134a (Fig. 4c), and LIFM-67 with numerous methyl groups can exhibit high adsorption capacity for R22 (11.2 mmol g^−1^) and R134a (11.1 mmol g^−1^) at 298 K and 1 bar despite possessing a small pore size (LIFM-66 is 16.2 Å, LIFM-66/67-mix is 15.6 Å, and LIFM-67 is 15.5 Å) and pore volume (LIFM-66 is 1.42 cm^3^ g^−1^, LIFM-66/67-mix is 1.30 cm^3^ g^−1^, and LIFM-67 is 1.27 cm^3^ g^−1^) [170]. In LIFM-66, R134a located in a pocket consisting of a Zr_6_-cluster, one ettc linker and two btb ligands, with the presence of strong C–H…F_FCF_ (2.76, 2.88, and 3.1 Å) interactions and weak vdW forces (3.28–3.92 Å). In LIFM-67, R134a located in a pocket surrounded by a Zr_6_-cluster and two ctta connectors, and there are strong C–H…F_FCF_ (2.52, 2.69, 2.77, 3.14, and 3.16 Å) and weak vdW forces (3.31–3.49 Å). These results further confirm that the introduction of methyl group enhances the interaction between the gas and the framework. The fluorine-functionalized MOFF-5 greatly enhances performance for adsorbing fluorocarbon/CFC gases, such as R12 (6.6 mmol g^−1^), R134a (5.4 mmol g^−1^), and CFC-113 (Cl_2_FC-CClF_2_, approximately 9.7 mmol g^−1^) [111, 186]. This feature is related to the high-polarity environment inside fluorinated cavities and the size of gas molecules. OMS-containing MOFs can adsorb fluorocarbon refrigerants at low pressure irrespective of pore geometry [77, 178, 187]. A study of R134a adsorption by mesoporous MOFs (Ni-MOF-74 members) with similar chemical properties, it demonstrated that the enthalpy of adsorption exceeded that reported for non-OMS MOFs (< 35 kJ mol^−1^) even though the increase in pore size resulted in the saturation of the open Ni^2+^ sites in Ni-MOF-74 with R134a (Ni-MOF-74 about 50 kJ mol^−1^; Ni-TPM, approximately 45 kJ mol^−1^) [179]. The ionic radii of metal sites on MOFs directly affect affinity with gas molecules. Adsorption studies of M-MOF-74 (M = Zn, Ni, Mg, Co) on refrigerants showed that Mg^2+^, which has small ionic radius, has strong C–F…M^+^ interactions that considerably increase the adsorption capacity of Mg-MOF-74 [175, 188].

MOFs with the highly tunable structure can obtain diverse topologies through the coordination of different metal nodes and ligands. Compared to structures with single and uniform pore size, MOFs with layered micro/mesoporous exhibit greater advantages in gas capture. Additionally, adjusting the pore size/shape, introducing defect sites to create additional pores, and introducing metal/nonmetal sites are all effective strategies for modulating gas adsorption isotherms.

### Separation of Fluorocarbon Refrigerants

Fluorocarbon refrigerants have different molecule size and configurations, polarity, and electronic structures, and separation based on a customized pore size or functional sites can be achieved using MOFs. UiO-66 is highly effective for separating and recovering binary (R22/R32, R32/R125) and ternary (R32/R125/R134a) fluorocarbon azeotropic mixtures [172]. These mixtures in decreasing order of adsorption strength are R134a, R125, R22, and R32 because highly polar gases usually have strong interactions with adsorbents. The large molecular size, molar mass, and high boiling points of the gases also promote vdW interactions (C–H…π and C–H…O interactions with phenyl in UiO-66) and gas–gas interactions. In the separation and recovery of R-401A (R32/R125 blend) and R-407F (R32/R25/R134a blend) by MOF-177, Cu-BTC, MIL-53, and ZIF-8, R125 and R134 can be preferentially adsorbed by all adsorbents at low pressure (< 3 bar) because of the enthalpy factor from a high degree of interaction between gases and MOFs. At high pressure (> 4 bar), gas adsorption may involve entropy factors, and thus, a selective shift to R32 with small molar volume or no selectivity occurs [174].

**Fig. 5:**
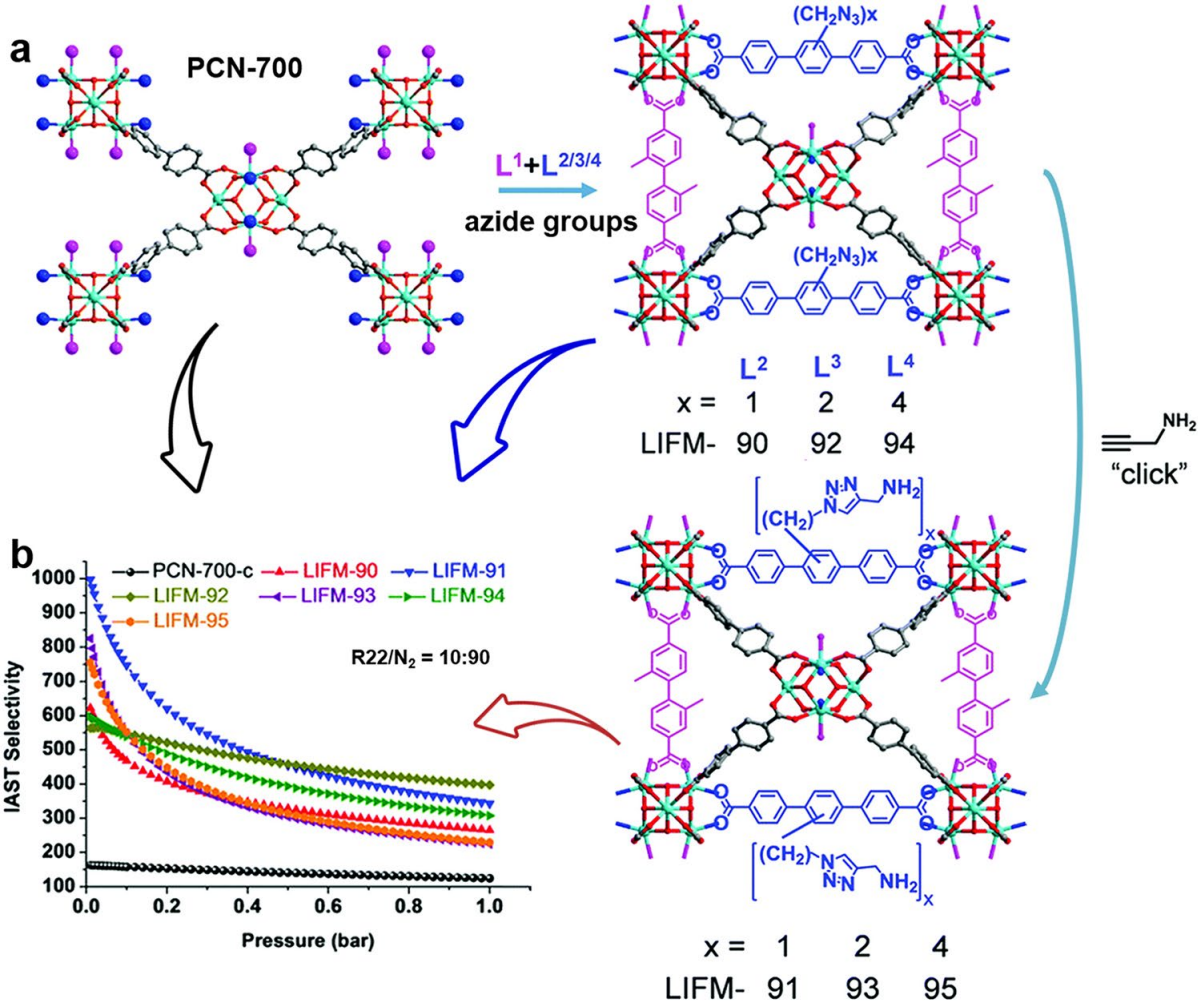
**a** Stepwise synthesis of LIFM-90–95 through the introduction of azide groups and click reaction using PCN-700 as a prototype. **b** IAST selectivity of LIFM-90–95 to R22/N_2_ at 273 K

Zr-MOF (DUT-67) composed of Zr_6_-nodes and 2,5-thiophenedicarboxylate linker has good separation ability for R22/R134a/CO_2_ [169]. The adsorption mechanism was analyzed by SCXRD and found that the Zr_6_-nodes with rich OH^−^/H_2_O group can selectively adsorb FC/CFCs through interactions between O–H…F or O–H…Cl and F/Cl atoms, which explained the corresponding high *Q*_st_ values of R22 (42.3 kJ mol^–1^) and R134a (49.3 kJ mol^–1^, *Q*_st_ of CO_2_ is 29.2 kJ mol^–1^). This is consistent with the adsorption position and adsorption energy trend (Δ*E*_R134a_ = 34.31 kJ mol^–1^, Δ*E*_R22_ = 26.31 kJ mol^–1^) obtained by GCMC simulation (the charges of R22/R134a and DUT were calculated using ESP and *Q*_eq_ methods, respectively [189, 190]; the force field parameters for Zr were obtained from the UFF, while others from the DREIDING force field [135, 191]) and DFT calculation (UFF). The pore space of PCN-700 as the prototype was divided by inserting azide groups as spacers after synthesis (LIFM-90/92/94), and interactions with guest molecules were enhanced by introducing 1,2,3-triazole functional groups (TAZ) through a click reaction with propylamine (LIFM-91/93/95), which effectively changes the pore environment and increases the performance of MOFs in separating R22/N_2_ (Fig. 5a) [173]. The initial selectivity (Fig. 5b) of LIFM-91/93/95 (the IAST of LIFM-91 reaches 996) bearing TAZ groups for R22/N_2_ is sixfold that of PCN-700-c (IAST, ~ 163). The introduction of polar functional groups in MOFs can enhance the interactions of MOFs with gas molecules through larger dipole or quadrupole moments or carbon–carbon double bonds, thus guiding the selective adsorption separation of gases [192]. The bifunctional (functional polar groups and OMS) LIFM-26 synthesized by connecting triangular prism Fe_3_(μ_3_-O) units with four chlorine atoms exhibits good separation performance for R22/N_2_ (IAST = 202) at 298 K and 1 atm, which is realized by its cooperation with the highly electronegative open Fe^II/III^ site and polar chlorine atoms [171]. However, the introduction of metal sites can produce strong electrostatic interactions with fluorocarbon refrigerants, which usually reduce adsorption selectivity and are not conducive to gas separation, especially when high product purity is required [77, 172, 193]. The flexible MOFs structure LIFM-28 with modified trifluoromethyl group was used as a prototype, and the reversible transition from narrow-pore to large-pore crystalline form was achieved by the precise installation of variable spacers with different functional groups after the synthesis, and the precise fine-tuning of the pore properties improved its adsorption selectivity for R22/N_2_ [192]. Compared to LIFM-28-lp (lp means large pore), the selectivity of LIFM-28–33 obtained by installing the spacer was improved by 4–7 times (IAST = 170–279, v/v = 10/90, 273 K). The abundance of fluorine atoms in the pores of the flexible–rigid MOFs (LIFM-100) promotes preferential adsorption of R22, and its selectivity up to 399.1 (298 K, v(R22)/v(N_2_) = 10/90) is higher than that of many reported MOFs structures (Table 4) [194]. It is worth mentioning that due to the existence of fluorine-containing functional groups, these MOFs structures have shown excellent thermal stability and chemical stability. Therefore, the combination of polar functional groups and the structural flexibility of MOFs will make the separation of complex fluorocarbon gas mixtures more controllable [195].

Fully understanding the behavior of MOFs and the different interactions and mechanisms involved in fluorocarbon refrigerants adsorption is the basis for developing new MOFs materials. Combining the differences of fluorocarbon refrigerant properties and the unique advantages of MOFs structure, the efficient separation of fluorocarbon refrigerant mixtures is within reach.

## Adsorption of VAs

Inhaled VAs, which are commonly used to induce and maintain general anesthesia in surgery, are fluorocarbons, and common VAs include sevoflurane, desflurane, and isoflurane [206–210]. The chemical structure and properties of VAs are similar to those of CFCs and HFCs which are potent greenhouse gases (Table 5) [211–214]. Unlike other F-gases, such as PFCs and CFCs, VAs are not restricted by legislation and international treaties due to “medical necessity” [215, 216].

**Table 5:** Physical properties of common VAs

Species [Refs.]	Chemical formula	Molecular weight	Boiling Point (K)	GWP (100 years)
Sevoflurane [206]	C_4_H_3_F_7_O	200.055	331.6	130
Isofluran [217]	C_3_H_2_ClF_5_O	184.49	321.5	510
Desflurane [218]	C_3_H_2_F_6_O	168.04	296.65	2540

**Fig. 6:**
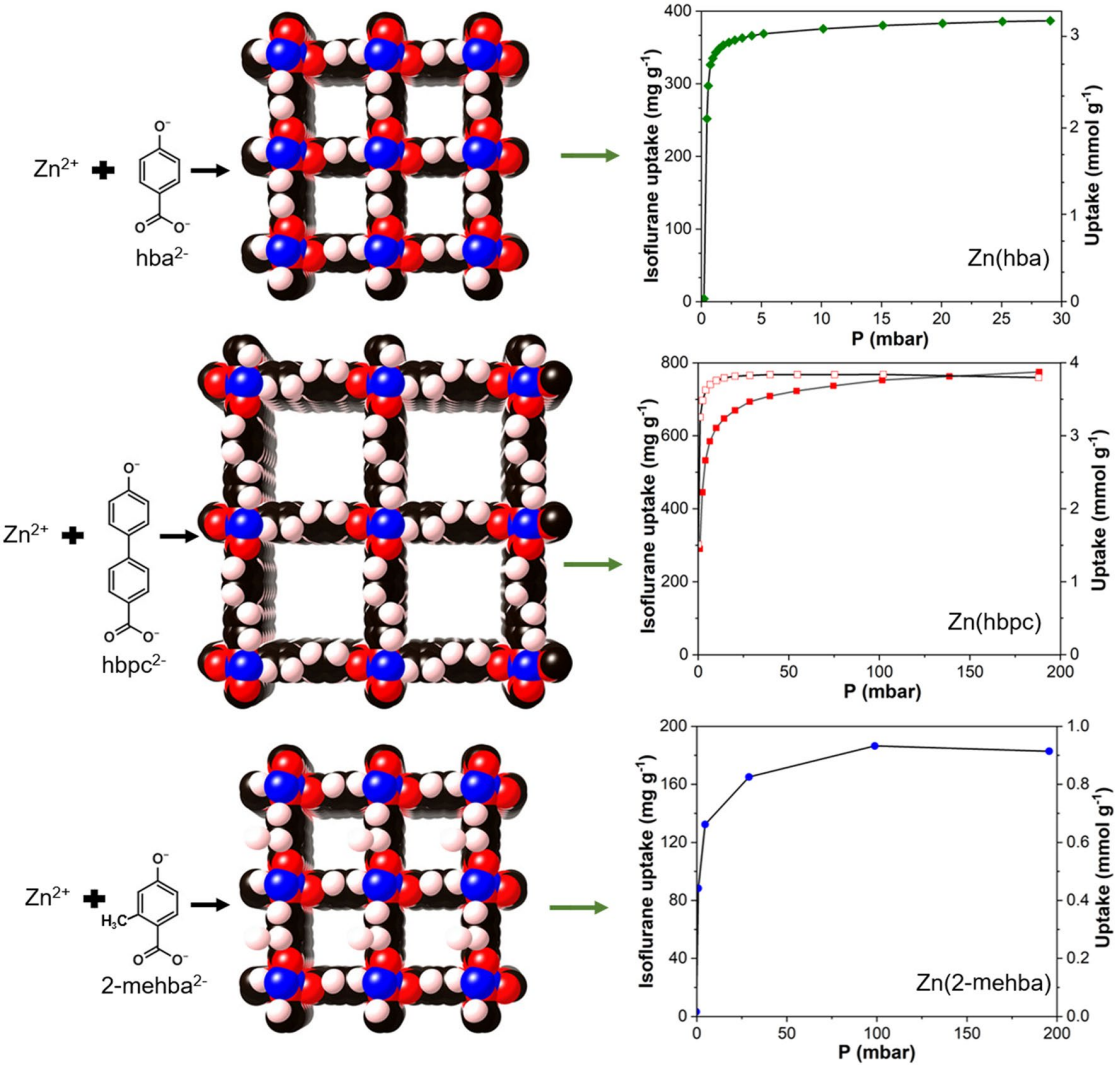
Network structure of ZnL (L = hba^2−^, hbpc^2−^, and 2-mehba^2−^), and the adsorption isotherms of isoflurane by Zn(hba), Zn(hbpc), and Zn(2-mehba) at 298 K

Zn(hba), Zn(hbpc), and Zn(2-mehba) have the same topological structure, and Zn(hbpc) (10 Å × 10 Å) obtained by using a long ligand has a larger pore size than Zn(hba) (6 Å × 6 Å), and saturated adsorption (4.2 mmol g^−1^) of isoflurane is twice that of Zn(hba) at 298 K (Fig. 6) [217, 219]. Zn(2-mehba) can be obtained by introducing methyl groups into the framework of Zn(hba), which has a small pore size due to the arrangement of methyl groups on its pore surface. Thus, it has low saturated adsorption performance for isoflurane (1.013 mmol g^−1^) but promotes the interactions of isoflurane with pore surfaces, which is favorable for storage. Chen et al. synthesized a porous fluorinated (1159 m^2^ g^−1^) MOFF-5 with a high surface area and good adsorption capacity (up to 73.4 ± 0.2% by weight) for common inhalational anesthetics, such as sevoflurane, enflurane, halothane, isoflurane, and methoxyflurane; the uptake of the anesthetics was quite fast, and saturated adsorption was reached within 130 s [111, 218]. However, the poor stability of MOFF-5 in water and under humid conditions limits its performance in capturing fluorocompounds. Mixed gases discharged by the anesthesia gas machine are usually accompanied by water vapor, and thus, enduring high moisture environments is the basis of capturing VAs by MOFs. MIL-101 shows excellent adsorption capacity (4.5 mmol g^−1^) for sevoflurane due to its high surface area (2041 m^2^ g^−1^) and abundant pore structure, and the adsorption capacity (3.9 mmol g^−1^) at 50% relative humidity only slightly decreases during single-component absorption [220, 221].

The promising application of MOFs in VAs capture provides new development possibilities for the healthcare industry. Placing small equipment using MOFs in medical settings or medical gas recovery systems can effectively reduce the risk to patients and medical staff exposed to VAs. Alternatively, an intelligent monitoring and control system can be developed by combining sensor technologies and artificial intelligence for the real-time monitoring and control of VAs emission, and automatic activation of MOFs device to capture VAs at concentrations exceeding the safety threshold, ensuring the safety of the medical environment.

## Adsorption of Other F-Gases

F-gases have potential applications in other important fields. The selective and late-stage introduction of fluoroethenyl and fluoroalkyl groups into drug-like molecules is the frontier in the field of organic synthesis, and common gaseous fluorinated commodities, such as trifluoropropene, vinylidene fluoride, trifluoromethyl iodide, and hexafluoropropene, are inexpensive and potential cornerstones for installing fluoroethenyl and fluoroalkyl groups [2, 222, 223]. They provide an entry point for fluorinated olefins, which are the important biological isomers of carbonyl groups, thus streamlining the synthesis of complex fluorinated molecules [224]. However, these gaseous fluorinated commodities are toxic, flammable, and environmentally unfriendly [225]. Therefore, the safe use of F-gases is critical for the synthesis of fluorinated molecules related to medicinal chemistry, agriculture, and biomedical imaging. The reversible adsorption of F-gases by porous solid materials allows for the treatment of F-gases as recyclable solid reagents, and MOFs can optimize the storage capacities and adsorption enthalpy (− ∆*H*_ads_) of guest molecules because MOFs are distinctively modular [226, 227]. MOFs containing OMS can reversibly bind synthetically relevant F-gases through strong metal–fluorine interactions [178, 228]. However, available data about the adsorption of gaseous fluorinated commodity by MOFs are few. The adsorption of F-gases, such as vinylidene fluoride, by 12 representative open metal-site MOFs is reversible, and M_2_(dobdc) containing high Lewis acidic Mg^2+^ sites exhibit high gravimetric capacities (7.95 mmol g^−1^, 34 wt%) and adsorption (− ∆ *H*_ads_ = 41 kJ mol^−1^) on vinylidene fluoride, demonstrating potential as an agent for transporting and storing controlled F-gases and enabling the development of high-throughput reactions for these gases [229].

## Technological Readiness Level

**Fig. 7:**
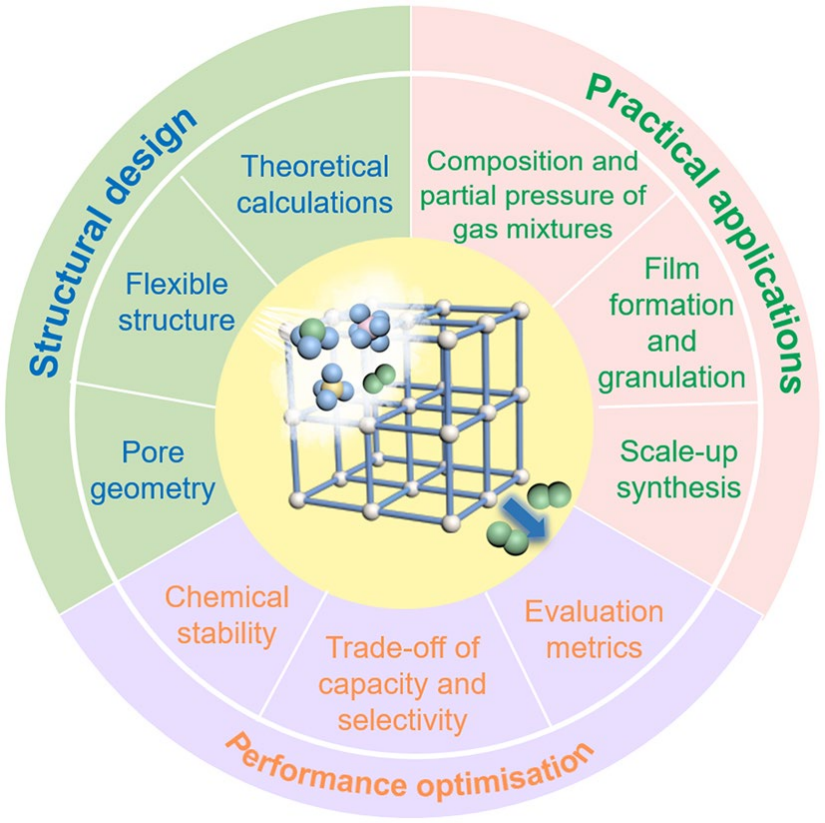
Key elements in the design of MOFs for the adsorption and separation of F-gases

MOFs adsorbents exhibit great potential in the field of trapping and separation of F-gases, but still face many unsolved challenges, and there also exist many directions worth exploring and trying to break through these bottlenecks (Fig. 7).

1. Breaking the trade-off between the selectivity and capacities of MOFs for F-gases is the key to designing novel MOFs. The preparation of MOFs with hierarchical porous structure provides opportunities for structural diversity and performance improvement of MOFs [230–232]. For example, a corrugated channel having small pores and large cavities can be designed, in which the small pores connect the large cavities and enhance adsorption selectivity, while the large cavities improve adsorption capacity. In addition, the structural flexibility of MOFs should be fully utilized, and dynamic structural changes induced by external stimuli not only are suitable for the separation of complex systems but also have high adsorption capacity in specific pressure regions.
2. The separation of F-gases by MOFs is based on differences in physical and chemical properties between different gas types. The typical example is the separation of SF_6_/N_2_, which is relatively easy due to the considerable difference in molecular size and polarity. However, when separating mixed gases of different PFCs species or fluorocarbon refrigerants, their highly similar physicochemical properties reduce the precision of identification when only the pore size of MOFs is considered. The ideal selectivity may be achieved by combining the dynamic diffusion regulation, thermodynamic equilibrium, and structural flexibility of MOFs, which requires a deliberate manipulation at the molecular or even atomic level of structural geometry, flexibility, and various intermolecular interactions.
3. In designing MOF structures, considering that most F-gases are inherently corrosive to MOFs is essential, which accelerate the oxidation or the other chemical reactions of metals and organic ligands, leading to structural collapse or performance degradation. Moreover, F-gases in practical production often contain water vapor and toxic or corrosive components (such as HF, CO, and NO_x_). Therefore, the high stability of MOFs is crucial to their separation performance, and the competitive adsorption of moisture and other gases should be considered.
4. In constructing design guidelines for MOFs that effectively separate F-gases mixtures, understanding of structure–activity relationships is crucial. The entire adsorption and separation processes should be monitored with the assistance of additional in situ characterization techniques. Mature characterization techniques, such as in situ single-crystal X-ray diffraction, powder X-ray diffraction, and neutron powder diffraction techniques, are used to detect the spatial binding sites of guest molecules. In situ infrared ray and in situ nuclear magnetic resonance techniques are employed to monitor structural changes in MOFs during adsorption. Different from the in situ characterization, the proposed operando enables the characterization of reactions under real working/operating conditions, offering opportunities for elucidating actual reaction mechanisms through real-time in situ characterization [233]. Three-dimensional electron diffraction and environmental transmission electron microscopy can allow for the direct observation of guests adsorbed by MOFs. Furthermore, interactions between MOFs and target gas molecules can be studied in depth with methods, such as computational chemistry and molecular dynamics simulations, for the accurate identification of adsorption mechanisms.
5. The composition and partial pressure of F-gases mixtures are crucial for evaluating the performance of MOFs. For example, SF_6_/N_2_ volume ratios of 0.1, 0.01, 0.002, and 0.0003 are common, and MOFs (such as SBMOF-1, Ni(ina)_2_) with high adsorption capacities in low-pressure regions are typically effective in separating SF_6_ from a mixture. IAST selectivity is often used as a reference metrics in evaluating separation performance, but it is calculated from a single-component adsorption isotherm and cannot fully represent selectivity in gas separation scenarios. In addition, the current gas flow rate for laboratory-scale breakthrough experiments is far below the industrial demand for time efficiency. Therefore, precise measurements are needed to refine and clarify the evaluation metrics and methods for MOFs separation performance in academic research.
6. In addition to high adsorption capacity and selectivity for F-gases, the realization of industrial applications also requires economic feasibility, excellent chemical stability, and ease of scalability in production [234, 235]. As can be seen from the compositions of MOFs listed in Tables 2 and 4, the raw materials for most MOFs are costly and not easily available. And the high-temperature and high-pressure equipment commonly used in its synthesis further increases both production costs and expenses related to safety precautions. Therefore, stable MOFs structures that can be obtained at room temperature using inexpensive raw materials can be selected as a foundation, and combine with the regulation of pore environment to make it have industrialization potential. MOFs such as ZJU-75a [235], MOF-303 [236], and Al-PyDC [237], which can realize industrial production in the field of olefin gas separation, provide reference for this. In addition, the formation of MOFs is also a crucial yet often overlooked step that affects industrialization [238–241]. Powdered MOFs will cause considerable drop in pressure and heat transfer problems in practical adsorption systems, which is unsuitable for direct integration into a commercial application [242]. Many traditional molding methods reported cannot be widely used in the synthesis of MOFs, and the optimization of its key parameters needs researchers to continue to strengthen their understanding of the characteristics of MOFs.

## Conclusion and Outlook

For controlling the emissions of F-gases, using MOFs for direct capture is one of the effective strategies for achieving climate objectives and fostering sustainable development in various industries. However, this strategy imposes specific demands on the adsorption capacities of MOFs. Consciously modulating energy and stereochemistry in the molecular structure of MOFs provides a unique platform for this purpose. Under the guidance of reticular chemistry, the adsorption capacities of MOFs for F-gases can be improved by using diverse topologies, ligand length, and by introducing defects. However, in practical applications, F-gases are often utilized as mixed gases to reduce energy consumption or meet the requirements of gas synthesis processes. Therefore, selectivity is another key indicator for separating and recovering target gases. Typically, to improve the adsorption selectivity of F-gases mixtures, the pore size of MOFs is often controlled to be close to the diameter of guest molecules. This approach strengthens vdW interactions between a host and guest. However, a narrow pore size usually results in a trade-off between adsorption capacity and selectivity. Metal or nonmetal functional sites that provide specific host–guest interactions for F-gases are crucial to offsetting this trade-off. Inhibiting the adsorption of other components by introducing sites of action is an ideal strategy for improving the separation performance of MOFs, and increasing the sites of action can further improve affinity for F-gases. However, this approach is not entirely satisfactory for the selective adsorption of PFCs/PFCs and fluorocarbon refrigerant blends with highly similar physicochemical properties. Flexible-robust MOFs can edit pores at the sub-angstrom level to realize precise regulation of pore environment, thus enabling the separation of F-gases with highly similar structures. At the same time, it overcomes the shortcomings of co-adsorption and low thermal/chemical stability of flexible MOFs. Flexible-robust MOFs can maintain its structural integrity at low pressure and show its significant plateau. More importantly, it has an adsorbate-dependent gas sorption isotherm, which is more suitable for molecular sieve components of complex systems. Numerous studies demonstrated the unique advantages of MOFs over other materials in the adsorption and separation of F-gases, but this emerging research field still faces many unresolved challenges, as well as a number of proposed directions worth exploring to address these bottlenecks. We encourage research on the adsorption and reuse of MOFs in other F-gases applications, which will reduce the negative impacts on the environment while promoting the effective utilization of potential resources for MOFs.

MOFs have great application potential for the actual adsorption and separation of F-gases and will be further developed through experiments and calculation research. The adsorption and separation strategies of F-gases in this review provide effective guidance for the design of other porous materials, such as organic frameworks and hydrogen-bonded organic frameworks. These materials have clear pore size and crystal structure, and thus, capturing gas mixtures by adjusting pore size geometry or introducing functional effective sites on the basis of reticular chemistry and advanced crystal engineering is feasible. In addition, the methods summarized and proposed in this review can be applied not only to MOFs but also to other systems, such as MOFs membrane, which provides a new solution for the design of the next-generation separation materials for F-gases.
